# Role of CCR1/5/7 in hepatocellular carcinoma: a study on prognostic evaluation, molecular subtyping, and association with immune infiltration

**DOI:** 10.18632/aging.205698

**Published:** 2024-03-28

**Authors:** Huasheng Huang, Chenlu Lan, Yongguang Wei, Jusen Nong, Xiwen Liao, Xinping Ye, Ganlu Deng, Tao Peng, Xin Zhou

**Affiliations:** 1 Department of Hepatobiliary Surgery, The First Affiliated Hospital of Guangxi Medical University, Nanning 530021, Guangxi Zhuang Autonomous Region, People’s Republic of China; 2 Guangxi Key Laboratory of Enhanced Recovery After Surgery for Gastrointestinal Cancer, Nanning 530021, People’s Republic of China; 3 Key Laboratory of Early Prevention and Treatment for Regional High Frequency Tumor (Guangxi Medical University), Ministry of Education, Nanning 530021, Guangxi Zhuang Autonomous Region, People’s Republic of China; 4 Department of Oncology, The First Affiliated Hospital of Guangxi Medical University, Nanning 530021, Guangxi Zhuang Autonomous Region, People’s Republic of China; 5 Guangxi Medical University, Nanning 530021, Guangxi Zhuang Autonomous Region, People’s Republic of China

**Keywords:** hepatocellular carcinoma, C-C chemokine receptor, transcriptome, prognosis, immune infiltration

## Abstract

This study aims to assess the prognostic value of the C-C motif chemokine receptor (CCR) gene family in hepatocellular carcinoma (HCC) and its relationship with immune infiltration and molecular subtypes of HCC. The evaluation of the GSE14520 dataset and TCGA database confirmed the prognostic significance of CCR. Building upon the correlation between CCR1, CCR5, and CCR7 and favorable prognosis, we further validated the prognostic importance of CCR1, CCR5, and CCR7 in ICGC database and an independent cohort from Guangxi autonomous region. Then, we constructed a risk prognosis model. Additionally, we observed significant positive correlations between CCR1, CCR5, and CCR7 and the infiltration of B cells, T cells, and macrophages in HCC. Subsequently, we conducted CCK assays, Transwell assays, and colony formation assays to evaluate the molecular biological functions of CCR1, CCR5, and CCR7. These experiments further confirmed that upregulation of CCR1, CCR5, and CCR7 can individually inhibit the proliferation, migration, and stemness of HCC cells. By analyzing the relationship between expression levels and tumor mutation frequency, we discovered that patients with high CCR1 expression were more likely to be classified as non-proliferative HCC. Similar conclusions were observed for CCR5 and CCR7. The association of CCR1, CCR5, and CCR7 with the molecular subtypes of HCC suggests that they may serve as intermediary molecules linking immune status and molecular subtypes in HCC. In summary, CCR1, CCR5, and CCR7 have the potential to serve as prognostic biomarkers for HCC and regulate HCC progression by influencing immune cell infiltration.

## INTRODUCTION

In the year 2020, 906,000 new cases of primary liver cancer were reported worldwide, ranking sixth for cancer incidence. Although liver cancer was the sixth most common malignant disease worldwide, it was the third cause of death resulting from malignancies [1, 2]. Hepatocellular carcinoma (HCC) accounts for approximately 90% of all primary malignant tumors of the liver [3, 4]. Cirrhosis of the liver, hepatitis B virus (HBV) infection, hepatitis C virus (HCV) infection, alcohol, nonalcoholic fatty liver disease (NAFLD), II diabetes, and obesity are the main high-risk factors for HCC [5, 6]. Overall HCC has led to great amount of suffering and has led to the decrease in the quality of life and a sharp reduction in survival time for patients, causing a tremendous economic burden to society. The situation of HCC in China is even more severe. HCC ranks fourth in incidence among all malignancies in China, with the resulting mortality ranking second [6, 7]. In some regions of China, such as Guangxi, high exposure to hepatitis B and aflatoxin has caused the incidence and mortality of hepatocellular carcinoma to locally rank first among all malignancies for four decades [7]. Even though the Tyrosine Kinase Inhibitor (TKI) represented by Sorafenib and Lenvatinib do extend the survival of some HCC patients, its overall therapeutic effect is not satisfactory [8, 9]. Furthermore, immune checkpoint inhibitor (ICI) alone does not produce satisfactory results in hepatocellular carcinoma [10, 11]. The results of clinical studies conducted during the past two years, in terms of the combination of TKI and ICI, seemed to offer hope to patients with advanced HCC [12]. The median progression-free survival (PFS) of patients administered Lenvatinib and Pabrizumab together has reached 9.7 months, and the 6-month and 12-month survival rates were 83.3% and 59.8%, respectively [13]. The results of the program are considered to be groundbreaking. Although breakthroughs have been made for the treatment of HCC, more effective treatment strategies need to be developed.

Chemokine receptors are known for their biological role in chemotaxis, target cell migration, and inflammation [14]. They are not only indispensable for all protective/ destructive immune and inflammatory activities, but also play a crucial role in the development and homeostasis of the human immune system [15, 16]. Due to their important role, chemokines are closely associated with multiple diseases, such as cancer, viral infections, inflammation, and autoimmune diseases. During recent decades, members of the chemokine system have been considered as potential targets in immunotherapy [17, 18]. Chemokines are a large class of chemotactic cytokines, with homologous receptors and chemokines receptors that are expressed in both tumor cells and stromal cells [19]. Given that chemokine receptors are involved in multiple aspects of cancer biology, their potential targets have been assessed in many preclinical studies and clinical trials. A recent study reported that chemokine receptor agonists could induce neutrophil extracellular traps that interfere with immune cytotoxicity [20]. Lesch et al. showed that CXCR6^+^ T cell adoptive therapy was effective in treating pancreatic cancer in mice [21]. In glioblastoma stem cell-like cells, the autocrine signaling of CCL5/CCR5 and CXCL12/CXCR4 enhance cell survival and self-renewal [22, 23]. In contrast, chemokines including CCL21, CXCL4, CXCL9, CXCL10, and CXCL11 have been shown to inhibit angiogenesis [24]. Monoclonal antibodies (anti-CCR4 mAb, Mogamulizumab) and chemokine receptor inhibitors (CXCR4 antagonist AMD3100) have been applied to hematologic malignancies in a clinical setting [25, 26]. The chemokine receptors have been grouped into subfamilies - CCR, CXCR, XCR and CX3CR – in terms of variations in their cysteine motifs. Based on the indications from the aforementioned studies, we conducted an analysis of the prognostic value and potential mechanisms of the CCR gene family.

## MATERIALS AND METHODS

### Functional annotation and pathway enrichment of the CCR genes

Functional annotation of the *CCR* genes was performed in terms of gene ontology (GO) and KEGG pathway using the Database for Annotation, Visualization and Integrated Discovery (DAVID) v6.8 [27, 28]. Then, the functional annotation clustering results were visualized in R studio using the packages, GOplot [29], Hmisc [30], and ggplot2 [31].

### Data sources and tissue specimen collection

The transcriptome sequencing matrix of the 212 HCC patients, which included 212 HCC tissues and 204 para-carcinoma tissues were used to obtain the corresponding prognostic data using the GSE14520 dataset obtained from the GEO database [32], and the para-carcinoma tissues of 8 patients were found to be missing. Transcriptome sequencing data of 370 HCC tissues and 50 para-carcinoma tissues were downloaded from TCGA database. Transcriptome data of 202 HCC tissues and 202 para-carcinoma tissues with complete survival data were obtained from the ICGC database. The liver tissues (paired HCC and para-carcinoma tissues) of 49 HCC patients at the First Affiliated Hospital of Guangxi Medical University were collected and then immersed in RNAstore Reagent (Tiangen, Beijing, China) within 30 minutes of collection. The tissue specimens were stored in a -80° C refrigerator. All 49 patients provided informed consent to participate in the study before the operation. This study was approved by the Ethics Committee of the First Affiliated Hospital of the Guangxi Medical University (Approval number: 2023-E485-01).

### Expression difference analysis, correlation analysis, and diagnostic efficiency

Student’s t test was used to analyze differences in the expression of the *CCRs* between HCC tumor tissues and para-carcinoma tissues. *P*<0.05 was considered to indicate statistical significance in the Student’s t test results. The correlation coefficient of *CCR* expression in HCC tissues was calculated in R software using the *corrplot* package. The receiver operating characteristic curve (ROC) was used to assess the diagnostic efficiency of the *CCRs*. If the area under curve (AUC) of the ROC curve exceeded 0.70, it was considered to be of satisfactory diagnostic efficacy.

### Survival analysis

The Kaplan-Meier method and Cox proportional hazards model were used to determine the survival analysis of HCC patients in the GSE14520 dataset based on the expression of *CCRs*. Bias created by differences in clinical characteristics on survival were adjusted for using the Cox proportional hazards model. The *CCRs* associated with the OS of HCC patients in the GSE14520 dataset were used to determine the combined effect of the survival analysis. Patients were assigned to groups based on the expression levels of multiple *CCRs*. The Kaplan-Meier plotter (https://kmplot.com/) is an online survival analysis website that is integrated with several databases [33]. It was used to further inspect the prognostic significance of the *CCRs* in the TCGA database. The Kaplan-Meier method was also applied to the survival analysis of the Guangxi cohort.

### Nomogram

A nomogram was constructed in R studio using the *foreign* package (Version 1.2.5033, R 3.6.2) in terms of clinical characteristics and the expression of *CCRs* [34]. Each index was scored by referring to its contribution based on prognosis, and the sum of the score was used as the risk score of each patient. The prediction probability of each individual was calculated through the functional transformation relationship between the total score and the occurrence probability of the terminal event. The bootstrap self-sampling method was used to verify the prediction efficiency of the nomogram.

### Prognostic signature construction

A prognostic signature was constructed based on the expression levels of the *CCRs* and prognosis related clinical parameters. Based on the regression coefficients and expression value of the *CCRs*, the risk score for each HCC patient was calculated: risk score = expression value of gene_1_ x β_1_+ expression value of gene_2_ x β_2_ +…+ expression value of gene_n_ x β_n_, where β was the regression coefficient derived from the multivariate Cox proportional hazards regression model. The Kaplan-Meier method was used to compare the outcome between high and low risk score groups. The time-dependent ROC curve was structured using the *survivalROC* package in R studio (Version 1.2.5033, R 3.6.2) to further evaluate prediction efficiency [35].

### Genome-wide exon mutation analysis of CCR genes

The genome-wide exon mutation data of TCGA cohort were downloaded from Genomic Data Commons (GDC) database, and were converted into mutation annotation format (MAF) by the *maftools* package in R studio (Version 1.2.5033, R 3.6.2) to explore the mutation characteristic of different expression level of CCR1, CCR5 and CCR7.

### Quantitative polymerase chain reaction (qPCR)

Total RNA was extracted from fresh tissues using the improved TRIzol method (HCC and para-carcinoma tissues) on samples from 49 HCC patients and was reversed transcribed into complementary DNA with Reverse transcription kit (Takara, USA). qPCR was used to quantitatively analyze the expression levels of *CCR1*, *CCR5,* and *CCR7* using Fast Start Universal SYBR Green Master (Roche, Mannheim, Germany). Primers for *CCR1*, *CCR5*, *CCR7,* and *GAPDH* (reference gene) were designed and synthesized by Sangon Biotech Company (Shanghai, China). The forward and reverse primer sequences of *CCR1*, *CCR5*, *CCR7* and *GAPDH* used are as follows:

GAPDH: forward 5′-TCAGCCGCATCTTCTTT-3′,

reverse 5′-CGCCCAATACGACCAAAT-3′

CCR1: forward 5′-CTGTGTCAACCCAGTGATCTAC-3′

reverse 5′-GAGGAAGGGGAGCCATTTAAC-3′

CCR5: forward 5′-GCAGCTCTCATTTTCCATACAG-3′

reverse 5′-GACACCGAAGCAGAGTTTTTAG-3′

CCR7: forward 5′-CATGCTCCTACTTCTTTGCATC-3′

reverse 5′-CACTGTGGCTAGTATCCAGATG-3′

### Immunohistochemistry (IHC)

The tissue sections were obtained from the Department of Pathology of the First Affiliated Hospital of Guangxi Medical University. IHC assay was performed using a universal two-step IHC kit (PV-9000, ZSGB-BIO, Biotech, Beijing, China) following the manufacturer’s protocols. The primary antibodies against CCR1 (DF2710, Affinity, Jiangsu, China), CCR5 (AF6339, Affinity, Jiangsu, China), and CCR7 (AF5293, Affinity, Jiangsu, China), as well as peroxidase-conjugated goat antirat IgG (ZB-2307, ZSGB-BIO, Beijing, China) were used to perform the IHC assay. Tumor sections were incubated overnight with primary antibodies at 4° C. The primary antibody titer was configured according to the IHC concentration recommended by the manufacturer (CCR1, 1:200; CCR5, 1:300; CCR7, 1:100).

### Gene set enrichment analysis (GSEA)

According to the median of *CCR* expression, the HCC patients in the GSE14520 dataset of TCGA were divided into high and low expression *CCR* groups. GSEA was used to explore whether there were statistical differences in the Molecular Signatures Database (MSigDB) c2 (c2.all.v7.0.symbols.gmt) between the genomes with high and low expression groups [36], by virtue of standardized enrichment scores and false detection rates as criteria to determine statistical significance. The significance threshold was set to P<0.05 and false discovery rate (FDR) to <0.25.

### Tumor-infiltrating immune cells

TIMER is a web server used for the comprehensive analysis of tumor-infiltrating immune cells [37] and was applied to determine the correlation between *CCR* genes and tumor-infiltrating immune cells. We mainly explored the correlation between *CCRs* and B cells, CD8^+^ T cells, CD4^+^ T cells, and macrophages. The correlation coefficient was used to evaluate the correlation between the expression level and the degree of cell invasion. The significance threshold was set to a correlation coefficient of >0.300 and *P*<0.05.

### Cell transfection

All transfection experiments in this study were performed using a transfection reagent on a Lipofectamine 3000 system (Invitrogen, USA) according to the manufacturer’s instructions. Three plasmids carrying the wild-type sequences of CCR1, CCR5, and CCR7, were purchased from Hanbio (Shanghai, China) to achieve upregulation of CCR1, CCR5, and CCR7 in hepatocellular carcinoma cells. The duration of the transfection experiments in this study was 24 hours.

### CCK-8 assays

1500 cells were placed in 96-well plates and 3 replicates were set up for each group. Then, 6 replicates were set up to examine cell viability at 6 different momentary points. The cells were incubated in a thermostat at 37° C in a 5% CO_2_ environment and cell viability was assayed every 24 hours. After mixing 10 μL of CCK-8 reagent (Dojindo, Japan) with 90 μL of DMEM, the resulting solution is the CCK-8 working solution. The cell culture medium is removed, and the CCK-8 working solution is added. Subsequently, the cells are incubated in a light-protected cell culture incubator for 1.5 hours. Finally, the absorbance at 450nm for each well is measured using a microplate reader.

### Transwell assays

After suspending the cells in serum-free culture medium, 50,000 cells were placed in the upper chamber of a culture well (Corning, USA). Subsequently, the chamber was placed in complete medium containing 10% fetal bovine serum. After 48 hours of incubation, the chamber was removed. Cells from the upper layer of the membrane were separated using a cotton swab. The chamber was then immersed in methanol for fixation for 20 minutes. Excess formaldehyde was washed away with water, followed by staining with crystal violet for 20 minutes. Finally, the chamber was washed three times with water to remove excess dye. Cells adhering to the lower surface of the culture well were observed under a microscope. The cell count in each field of view was recorded, and the average cell count from 5 random fields of view was calculated to represent the number of cells that crossed the permeable membrane per unit area.

### Colony formation assays

Firstly, place 500 cells in each well, ensuring that each well contains 2 ml of complete culture medium to prevent evaporation during the experiment. Incubate the culture plates in a 37° C, 5% CO2 incubator for 2 weeks. Perform the experiment with three replicates for each group. After the two-week incubation, viable cells should have adhered and formed colonies. Remove the residual culture medium, wash the cells twice with sterile PBS, and then fix the cells with 4% paraformaldehyde for 20 minutes. Afterward, wash the cells twice with sterile PBS and add 1 ml of crystal violet for staining for 10 minutes. Wash away excess crystal violet dye, and the cells can be observed and counted.

### Statistical analysis

Student’s t-test was used to compare differences in the expression between the HCC group and para-carcinoma group. The Kaplan-Meier method along with the log-rank test and Cox proportional hazards model was respectively applied for the survival analysis. ROC analysis was performed to assess diagnostic efficiency. Statistical calculations were performed using SPSS 22.0 or R studio (Version 1.2.5033, R 3.6.2) software, except for GSEA. Statistical analysis of GSEA data was performed using GSEA v4.0.3 software. Statistical significance was achieved when *P*<0.05 in the Student’s t-test, ROC, log-rank test and Cox proportional hazards model. The hazards ratio is shown along with a 95% confidence interval.

### Availability of data and material

The datasets used and/or analyzed during the current study are available from the corresponding author on reasonable request.

## RESULTS

### Functional annotation and pathway enrichment results of the CCR genes

**Figure 1:**
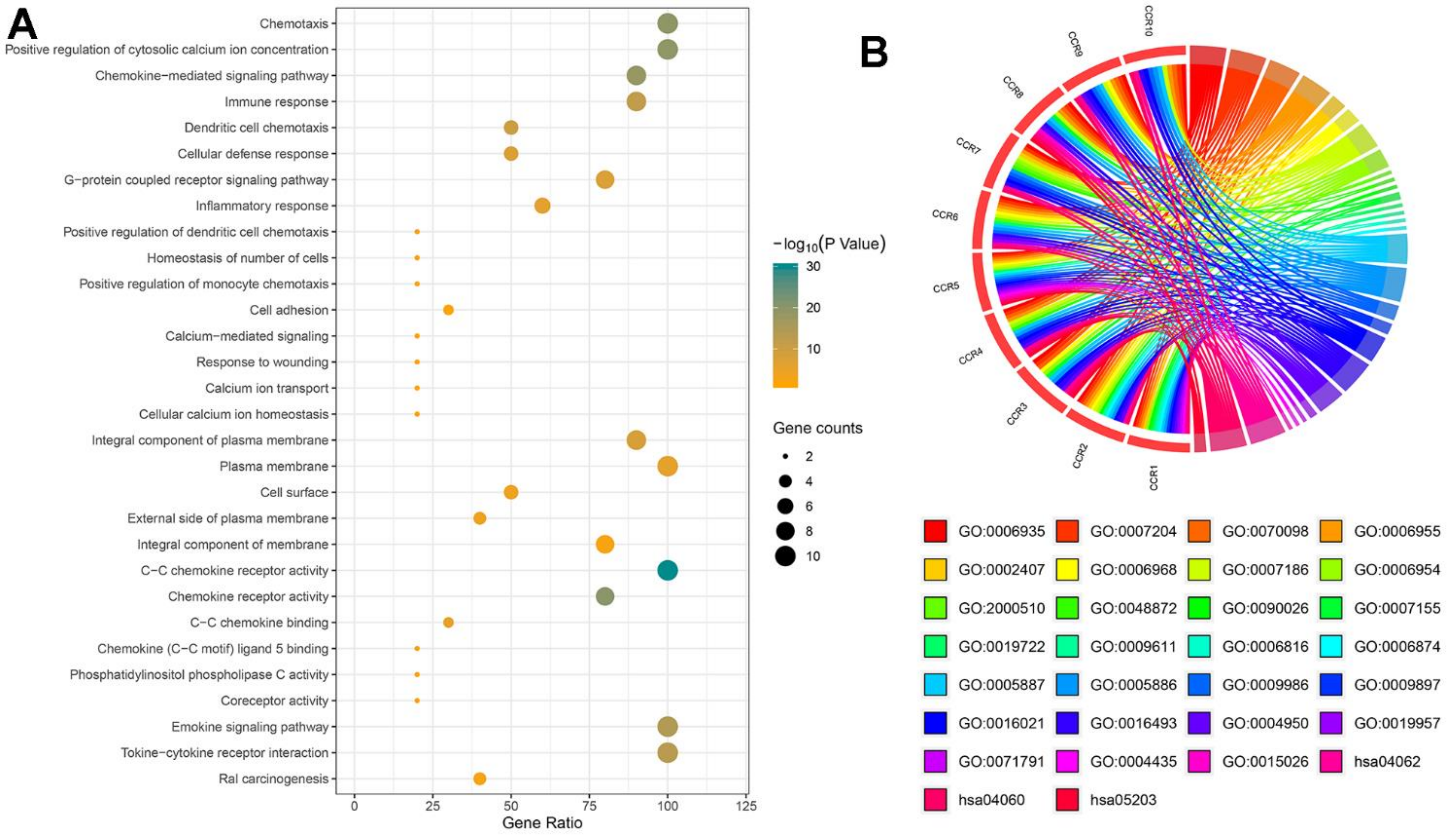
**Bioinformatics-based results from DAVID.** (**A**) The pathways, molecular functions, biological processes, and cellular components in which *CCRs* are enriched; (**B**) details of *CCRs* corresponding to specific pathways, molecular functions, biological processes and cellular components. GO, Gene ontology.

The DAVID database was used to analyze the biological functional annotation of the CCR family of genes. The results of the biological functional annotation are presented as a bubble chart and chord chart. The gene functional enrichment analysis showed that the biological function of the CCR family of genes was mainly enriched in chemotaxis, regulation of cytosolic calcium ion concentration, chemokine-mediated signaling pathway, immune response, dendritic cell chemotaxis, and cellular defense response (Figure 1A). The -log(P-value) is indicated by the color of the bubbles. The correspondence between *CCRs* and GO terms is shown using a chord chart (Figure 1B). The details of the enriched Gene Ontology (GO) terms in molecular function (MF), biological process (BP), and cellular component (CC) categories and KEGG pathway for CCR genes from DAVID database are displayed in Supplementary Table 1.

### Expression of the *CCRs* in the HCC and para-carcinoma tissues

**Figure 2:**
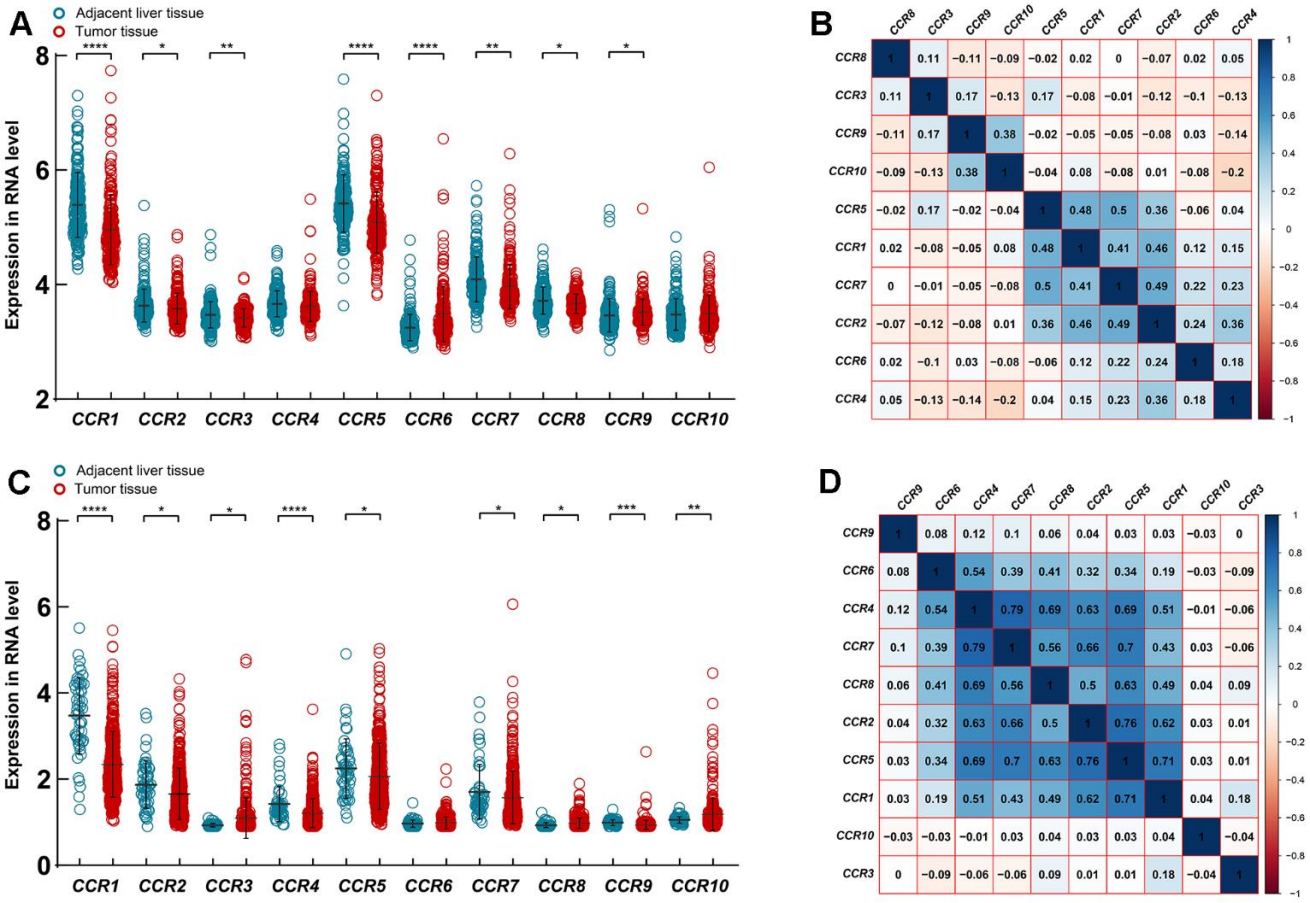
**Expression of *CCRs* in HCC and para-carcinoma tissues.** (**A**) Expression level of *CCRs* between HCC and para-carcinoma tissues in GSE14520; (**B**) Matrix graphs of Pearson correlations for *CCRs* in GSE14520; (**C**) expression level of *CCRs* between HCC and para-carcinoma tissues in TCGA database; (**D**) Matrix graphs of Pearson correlations for *CCRs* in TCGA database. * P<0.05, ** P<0.01, *** P<0.001, **** P<0.0001.

Due to the partial absence of paracancer tissue in the GSE14520 dataset, unpaired student’s t-test was used to analyze expression differences. The GSE14520 dataset showed that the expression levels of *CCR1*, *CCR2*, *CCR3*, *CCR5*, *CCR7,* and *CCR8* in the HCC tissues were significantly lower than that of the para-carcinoma liver tissues, whereas the expression of *CCR6* and *CCR9* was higher in the HCC tissues (Figure 2A). CCR4 and CCR10 are the only two members of the CCR family that show no difference in expression levels between HCC and para-carcinoma liver tissues. Expression correlation analysis between any two members of the CCR family showed that there were strong correlations among the expression of *CCR1*, *CCR2*, *CCR5*, and *CCR7* in HCC (Figure 2B).

Additionally, the expression characteristics of the CCR family genes were further evaluated using the TCGA LIHC dataset. The expression levels of *CCR1*, *CCR2*, *CCR4*, *CCR5*, *CCR7,* and *CCR9* were significantly lower in HCC tissues, whereas the expression levels of *CCR3*, *CCR8,* and *CCR10* were significantly higher in HCC tissues (Figure 2C). The expression correlation analysis indicated that there were expressional correlations between *CCR1*, *CCR2*, *CCR4*, *CCR5*, *CCR6, CCR7,* and *CCR8* in HCC (Figure 2D).

### Diagnostic significance of CCRs in HCC

After a preliminary exploration of the expression characteristics of members of the CCR gene family in HCC and para-carcinoma liver tissues, we assessed the efficiency of these genes as diagnostic markers of HCC using the area under the ROC curve (AUC). In the GSE14520 cohort, *CCR1* (AUC=0.731, Supplementary Figure 1A) and *CCR5* (AUC=0.714, Supplementary Figure 1E) were observed to produce reasonable diagnostic performance in HCC, while the diagnostic significance of the other CCR family members (Supplementary Figure 1B–1D, 1F–1J) were not satisfactory. In TCGA cohort, *CCR1* (AUC=0.833, Supplementary Figure 1K) and *CCR9* (AUC=0.835, Supplementary Figure 1S) were exhibited satisfactory diagnostic performance in HCC, while the diagnostic efficiency of the other CCR family members (Supplementary Figure 1L–1R, 1T) were not satisfactory.

### Survival analysis results of the GSE14520 dataset and TCGA

Whole-transcriptome microarray data, prognostic data, and clinical information on the 212 HCC patients were obtained from the GSE14520 dataset. The relationship between clinical factors and prognosis were investigated for bias correction through subsequent survival analyses of the *CCR* genes. The baseline information and univariate Cox regression results on the 212 HCC patients is presented in Supplementary Table 2. In the GSE14520 dataset, tumor size (*P*=0.002; HR=1.975, 95% CI:1.274-3.060), cirrhosis (*P*=0.025; HR=4.335, 95% CI: 1.065-17.638), BCLC stage (*P*<0.001; HR=18.993, 95% CI: 4.419-81.632), TNM stage (*P*<0.001; HR=3.425, 95% CI: 2.171-5.405), and AFP (*P*=0.049; HR=1.546, 95% CI: 1.002-2.385) were associated with the OS of HCC, while gender (*P*=0.018, HR=2.142, 95% CI: 1.120-4.100), TNM stage (*P*<0.001; HR=2.279, 95% CI: 1.517-3.423), and BCLC stage (*P*<0.001; HR=6.163, 95% CI: 2.477-15.333) were associated with the RFS of HCC.

The relationships between the *CCR* family of genes and RFS were explored using the GSE14520 dataset and TCGA. In the GSE14520 cohort, none of the *CCR* genes were observed to be associated with the RFS of patients in HCC, neither using the Kaplan-Meier method nor the Cox proportional hazards model (Table 1 and Supplementary Figure 2A–2J). In TCGA cohort, *CCR1* (*P*=0.023), *CCR2* (*P*<0.001), *CCR4* (*P*=0.007), *CCR5* (*P*<0.001), *CCR6* (*P*<0.001), *CCR7* (*P*<0.001), *CCR8* (*P*=0.029)*,* and *CCR9* (*P*=0.015) were observed to be associated with the RFS of the HCC patients (Supplementary Figure 2K, 2L, 2N–2S), while no prognostic significance was found for *CCR3* and *CCR10* (Supplementary Figure 2M, 2T).

**Table 1:** Prognosis significance evaluation for patients in HCC in terms of expression of *CCRs*.

**Gene expression**	**Patients (n=212)**	**RFS**							**OS**					
		**No. of event**	**MRT (months)**	**Crude HR (95% CI)**	**Crude *P***	**Adjusted HR (95% CI)**	**Adjusted *P* £**		**No. of event**	**MST (months)**	**Crude HR (95% CI)**	**Crude *P***	**Adjusted HR (95% CI)**	**Adjusted *P* §**
*CCR1*														
Low	106	62	38	1		1			46	NA	1		1	
High	106	54	52	0.827 (0.574-1.191)	0.307	0.763 (0.525-1.107)	0.154		36	NA	0.747 (0.483-1.156)	0.189	0.623 (0.394-0.987)	0.044
*CCR2*														
Low	106	61	42	1		1			44	NA	1		1	
High	106	55	47	0.849 (0.590-1.223)	0.379	0.872 (0.603-1.263)	0.470		38	NA	0.787 (0.509-1.214)	0.277	0.724 (0.458-1.144)	0.167
*CCR3*														
Low	106	63	36	1		1			47	NA	1		1	
High	106	53	55	0.810 (0.562-1.168)	0.257	0.90 (0.634-1.335)	0.661		35	NA	0.738 (0.476-1.143)	0.172	0.903 (0.573-1.422)	0.659
*CCR4*														
Low	106	63	33	1		1			44	NA	1		1	
High	106	53	54	0.727 (0.505-1.049)	0.086	0.713 (0.492-1.034)	0.074		38	NA	0.802 (0.519-1.238)	0.317	0.805 (0.515-1.258)	0.341
*CCR5*														
Low	106	64	29	1		1			48	61	1		1	
High	106	52	58	0.686 (0.476-0.991)	0.043	0.703 (0.484-1.022)	0.065		34	NA	0.602 (0.388-0.935)	0.022	0.587 (0.373-0.923)	0.021
CCR6														
Low	106	54	52	1		1			36	NA	1		1	
High	106	62	36	1.241 (0.861-1.787)	0.245	1.165 (0.806-1.684)	0.416		46	NA	1.359 (0.878-2.102)	0.167	1.208 (0.753-1.940)	0.434
*CCR7*														
Low	106	63	29	1		1			48	NA	1		1	
High	106	53	53	0.733 (0.508-1.056)	0.094	0.824 (0.568-1.194)	0.306		34	NA	0.599 (0.386-0.930)	0.021	0.621 (0.395-0.977)	0.039
*CCR8*														
Low	106	53	52	1		1			39	NA	1		1	
High	106	63	44	1.200 (0.832-1.731)	0.327	1.062 (0.731-1.544)	0.751		43	NA	1.117 (0.724-1.724)	0.616	0.937 (0.601-1.463)	0.775
*CCR9*														
Low	106	60	44	1		1			46	NA	1		1	
High	106	56	47	0.811 (0.612-1.269)	0.496	0.944 (0.651-1.369)	0.763		36	NA	0.765 (0.495-1.184)	0.228	0.810 (0.521-1.260)	0.349
*CCR10*														
Low	106	60	41	1		1			44	NA	1		1	
High	106	56	52	0.928 (0.644-1.336)	0.687	0.885 (0.610-1.283)	0.519		38	NA	0.873 (0.565-1.348)	0.538	0.821 (0.526-1.282)	0.385

Notes: £ in RFS of patients in HCC adjusted for tumor size, gender, TNM stage and BCLC stage; § in OS of patients in HCC adjusted for tumor size, cirrhosis, BCLC stage, TNM stage and AFP.

Abbreviation: CCR, C-C chemokine receptor; RFS, recurrence-free survival; OS, overall survival; NO, number; MRT, median recurrence time; HR, hazard ratio; CI, confidence interval; MST, median survival time.

**Figure 3:**
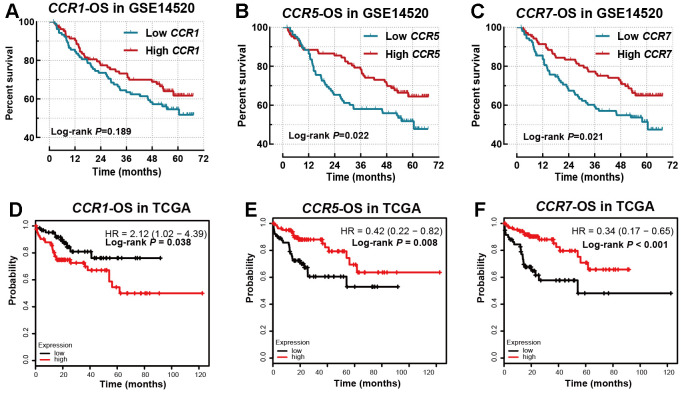
**Survival analysis for OS in GSE14520 and TCGA database.** (**A**) *CCR1* in GSE14520 dataset; (**B**) *CCR5* in GSE14520 dataset; (**C**) *CCR7* in GSE14520 dataset; (**D**) *CCR1* in TCGA database; (**E**) *CCR5* in TCGA database; (**F**) *CCR7* in TCGA database.

Then, we evaluated the relationship between CCR family members and OS of HCC patients in the GSE14520 and TCGA dataset. The prognostic significance of *CCR1* gene did not show prognostic significance for OS (*P*=0.189, Table 1 and Figure 3A) in the univariate survival analysis using the Kaplan-Meier method. However, it was observed to be associated with OS using the Cox proportional hazards model after adjusting for clinical factors (adjusted *P*=0.044, Table 1). *CCR5* (*P*=0.022, adjusted *P*=0.021, Table 1 and Figure 3B) and *CCR7* (*P*=0.021, adjusted *P*=0.039, Table 1 and Figure 3C) were both found to be significantly correlated with the OS of the HCC patients in the GSE14520 cohort, using either the Cox proportional hazards model or the Kaplan-Meier method. However, other members of the *CCR* gene family were not found to be associated with the OS of the HCC patients in the GSE14520 dataset (Supplementary Figure 3A–3G). Subsequently, the relationship between CCR1, CCR5, and CCR7 with clinical prognosis was analyzed (Supplementary Tables 3–5). CCR5 was found to be associated with tumor diameter, since a smaller proportion of HCC patients with tumors larger than 5 cm were included in the high CCR5 expression group than in the low CCR5 expression group. This indicates a negative correlation between CCR5 and tumor load.

*CCR1* (Figure 3D, *P*=0.044), *CCR5* (Figure 3E, *P*=0.044) and *CCR7* (Figure 3F, *P*=0.044) were also observed to be associated with the OS of TCGA cohort. In addition, CCR2, CCR3, and CCR4 were found to be associated with OS (Supplementary Figure 3H–3J), while *CCR6*, *CCR8*, *CCR9,* and *CCR10* did not show any prognostic significance (Supplementary Figure 3K–3N).

### Nomogram and prognostic signature

**Figure 4:**
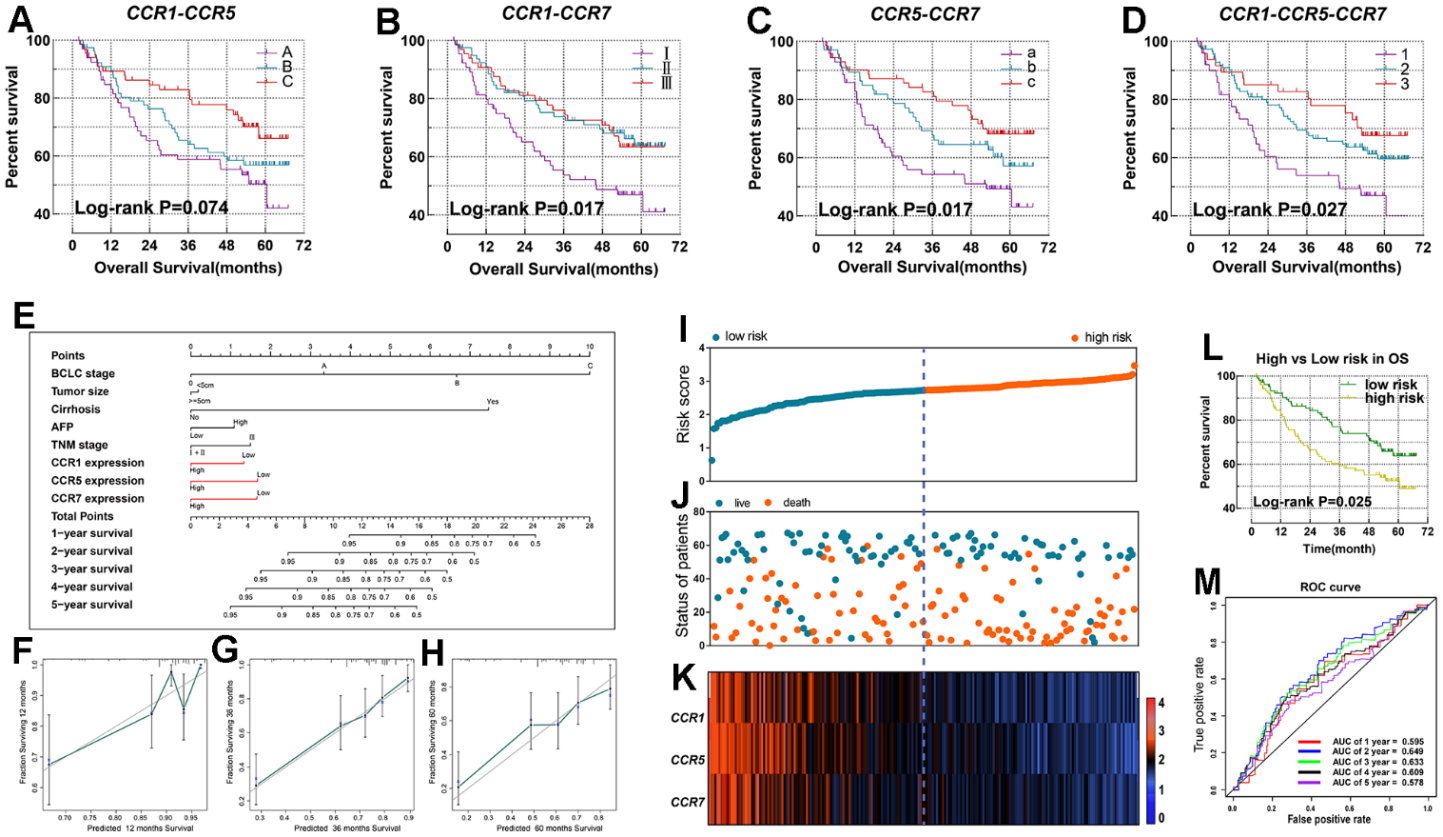
**Nomogram and the prognostic signature constructed in GSE14520 in terms of *CCR1*, *CCR5* and *CCR7*.** (**A**–**D**) Combined effect survival analysis for OS on the basis of *CCR1*, *CCR5* and *CCR7*; (**E**) nomogram; (**F**–**H**) verification model for nomogram in 1-, 2- and 3-year OS respectively; (**I**) risk score plot; (**J**) survival status scatter plot; (**K**) heat map of the levels of expression of *CCR1*, *CCR5* and *CCR7* in low- and high-risk groups; (**L**) Kaplan-Meier curves for low- and high-risk groups; (**M**) receiver operating characteristic curve for predicting 1-, 2- and 3-year survival in HCC patients by risk score.

Based on the prognostic significance of CCR1, CCR5, and CCR7, we performed a combined effect survival analysis, and created a nomogram and prognostic signature based on GSE14520 data, to optimize our discovery and produce a better predictive prognostic model for HCC patients. The combined analysis of *CCR1* and *CCR5* in HCC showed that patients in the low *CCR1* and *CCR5* expression group showed the best outcome (Figure 4A). Similarly, in other combined analyses, patients in group III, group c, and in group 3 all exhibited comparatively longer survival (Figure 4B–4D). The grouping protocols and outcomes are listed in Table 2. We observed that differences between the best and worst groups were more significant in the combined analysis than in the single gene survival analysis.

**Table 2:** Joint effects analysis of *CCR1*, *CCR5* and *CCR7* in GSE14520.

**Group**	***CCR1***	***CCR5***	***CCR7***	**Patients**	**NO. of event**	**MST(Months)**	**Crude HR (95% CI)**	**Crude P**	**Adjusted HR (95% CI)**	**Adjusted P δ**
A	Low	Low		67	31	61	1		1	
B	Low	High		78	32	NA	0.777(0.474-1.274)		0.920(0.547-1.547)	
	High	Low								
C	High	High		67	19	NA	0.518(0.293-0.918)	0.074	0.445(0.245-0.808)	0.008
I	Low		Low	66	34	47	1		1	
II	Low		High	80	26	NA	0.526(0.315-0.877)		0.437(0.255-0.748)	
	High		Low							
III	High		High	66	22	NA	0.541(0.315-0.926)	0.017	0.491(0.285-0.846)	0.010
a		Low	Low	72	35	53	1		1	
b		Low	High	68	26	NA	0.659(0.396-1.095)		0.588(0.350-0.988)	
		High	Low							
c		High	High	72	21	NA	0.468(0.272-0.805)	0.017	0.473(0.271-0.824)	0.008
1	Low	Low	Low	51	26	47	1		1	
2	Low	Low	High	113	42	NA	0.592(0.363-0.967)		0.586(0.352-0.976)	
	High	Low	Low							
	Low	High	Low							
	Low	High	High							
	High	High	Low							
	High	Low	High							
3	High	High	High	48	14	NA	0.450(0.235-0.862)	0.027	0.450(0.233-0.869)	0.017

Notes: δ in OS of patients in HCC adjusted for tumor size, cirrhosis, BCLC stage, TNM stage and AFP.

Abbreviation: CCR, C-C chemokine receptor; NO, number; MST, median survival time; HR, hazard ratio; CI, confidence interval.

We established a nomogram and a prognosis signature based on the expression levels of *CCR1*, *CCR5,* and *CCR7* in the GSE14520 dataset. In the nomogram, the length of the corresponding line segment of each variable represents its degree of contribution to prognosis. The parameter with the highest prognostic contribution was BCLC stage, followed by the degree of cirrhosis. The contribution of *CCR1*, *CCR5,* and *CCR7* for the prediction of prognosis was similar (Figure 4E). We evaluated the predictive power of the histogram by matching the degree between the training group and the validation group. In the nomogram created using GSE14520 data, there was a high degree of superposition between the self-validation cohort (red line) and the training group (gray line) in predicting the 1-, 3 -, or 5-year prognosis (Figure 4F–4H).

The risk score formula of the prognosis signature in the GSE14520 dataset was: risk score = expression value of *CCR1* x -0.278 + expression value of *CCR5* x -0.348 + expression value of *CCR7* x -0.306. A total of 212 patients with HCC in the GSE14520 dataset were classified as the high-risk group or low-risk group. Patients were ranked using the risk score from left to right (Figure 4I, 4K) and we observed that patients in the high-risk group had a higher concentration of individuals who reached a terminal event within a short duration (Figure 4J). The difference between the high and low risk groups in OS was statistically significant (*P*=0.025, Figure 4L). Additionally, the ROC curve revealed that the prognostic signature showed good performance in predicting the 1-, 2-, 3-, 4-, and 5-year outcomes (Figure 4M).

### CCR1, CCR5, and CCR7 expression was associated with mutations of TP53 and CTNNB1

**Figure 5:**
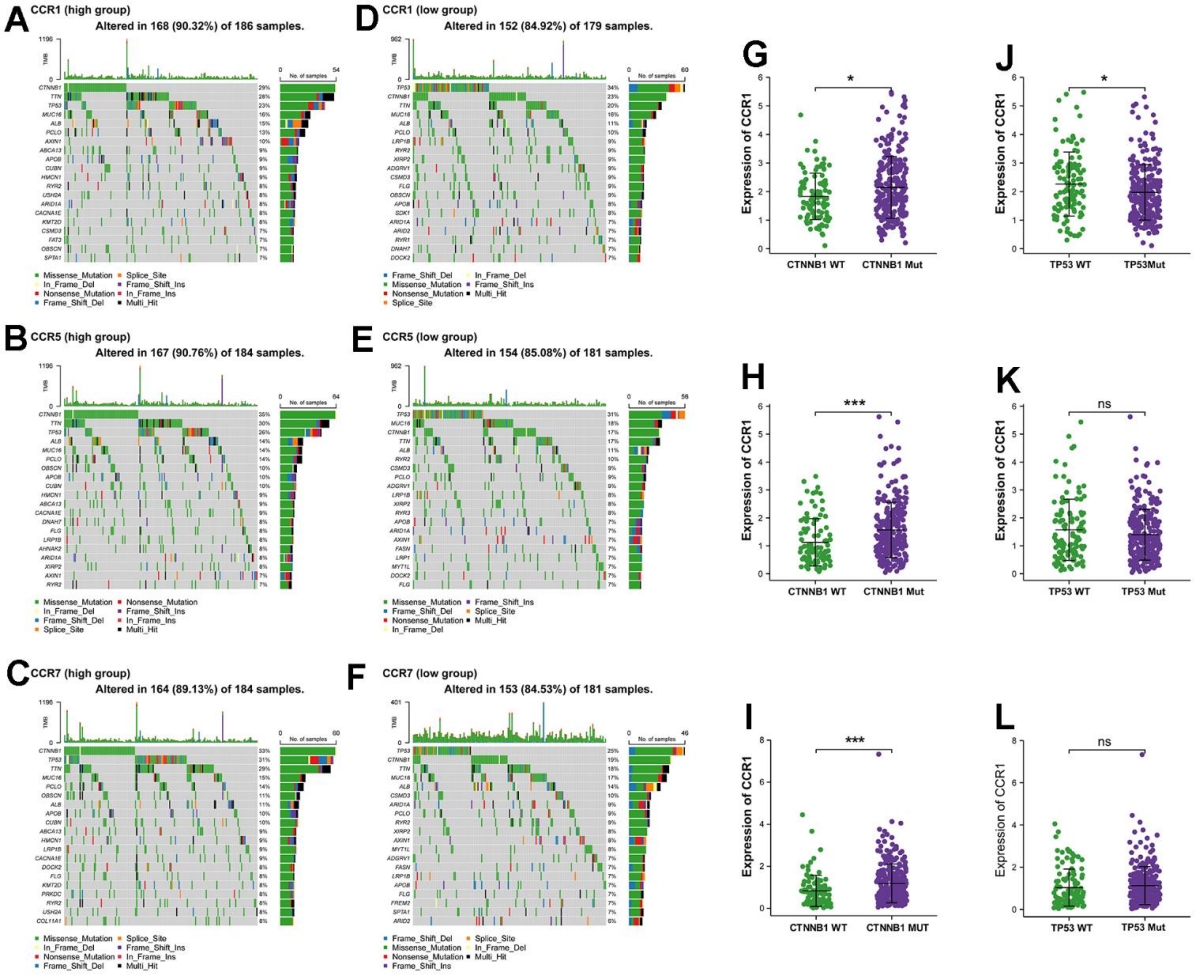
**The genome-wide exon mutation characteristics of CCR1, CCR5 and CCR7 expression group.** (**A**–**F**) Waterfall plot of genome-wide exon mutation of TCGA cohort in different expression levels of *CCR1*, *CCR5* and *CCR7*. (**G**–**L**) Expression levels of *CCR1*, *CCR5* and *CCR7* in TCGA cohort based on the mutant and wild-type of CTNNB1 and TP53. * P<0.05; *** P<0.001, ^ns^ no significance.

HCC is typically classified into two types based on mutation characteristics. Those with TP53 mutations belong to the proliferative type, indicating a poorer prognosis, while those with CTNNB1 mutations belong to the non-proliferative type, indicating a relatively better prognosis. In the analysis in TCGA database, among all the exon, the mutation frequency of CTNNB1 was the highest in patients with high expression of CCR1, CCR5 and CCR7 (Figure 5A–5C), while the TP53 mutation ranked first in the patients with low expression of CCR1, CCR5 and CCR7 (Figure 5D–5F).

Subsequently, we categorized the HCC patients in the TCGA cohort into two groups based on the presence or absence of CTNNB1 mutations. In the CTNNB1 mutation group, the expression levels of CCR1, CCR5, and CCR7 were significantly higher compared to the non-mutation group (Figure 5G–5I). Additionally, we also divided the HCC patients in the TCGA cohort into two groups based on the presence or absence of TP53 mutations. It was observed that the expression levels of CCR1 were significantly lower in the TP53 mutation group compared to the non-mutation group (Figure 5J). These analyses revealed a correlation between the CCR gene family and the molecular subtypes of HCC defined by gene mutations, suggesting that CCR1, CCR5, and CCR7 may serve as bridging molecules connecting the molecular subtypes of HCC and immune infiltration.

### Validation of the clinical significance of CCR1, CCR5, and CCR7 in the Guangxi cohort and ICGC dataset

**Figure 6:**
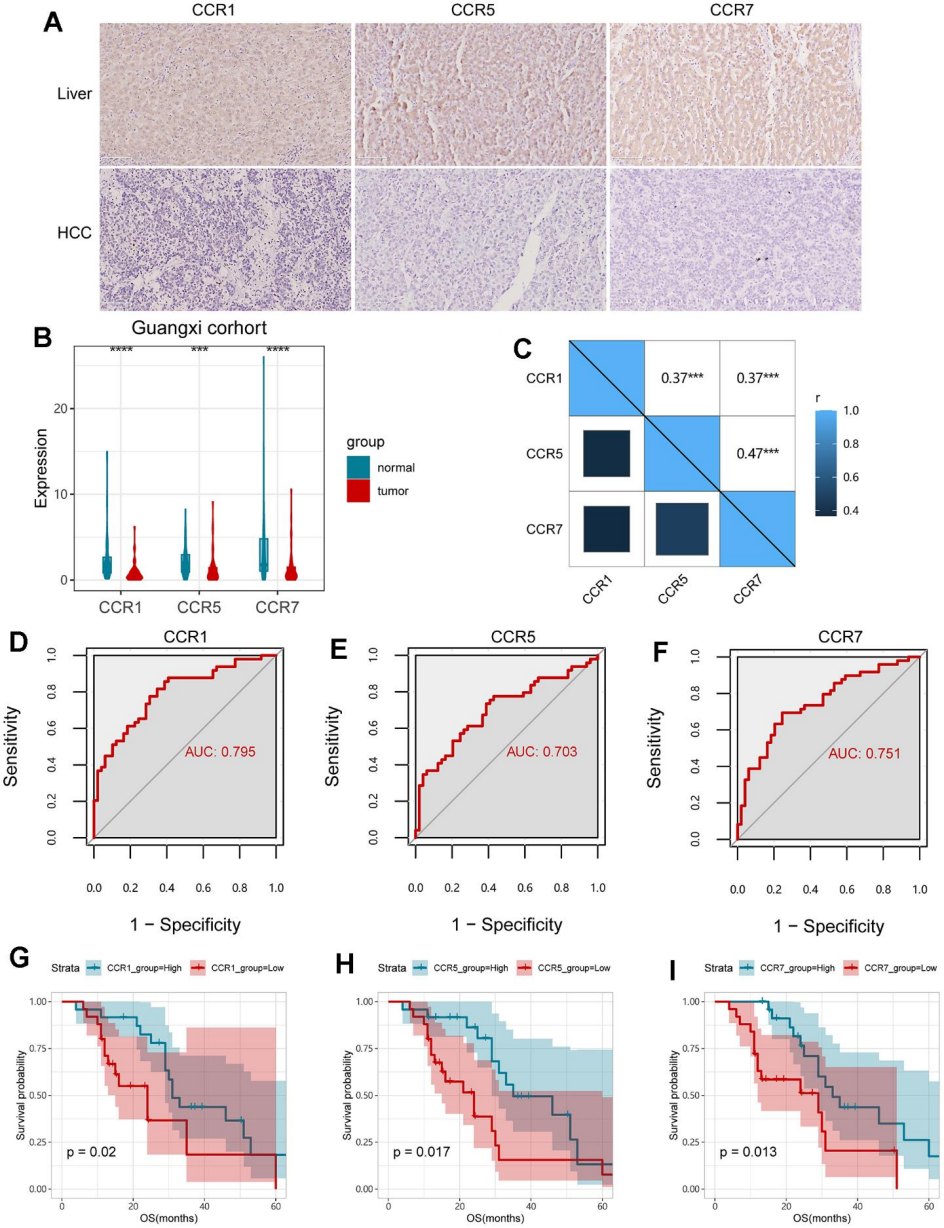
**Validation of *CCR1*, *CCR5* and *CCR7* in Guangxi cohort.** (**A**) Expression of *CCR1*, *CCR5* and *CCR7* in HCC and para-carcinoma live tissues detected with IHC assay; (**B**) expression of *CCR1*, *CCR5* and *CCR7* in HCC and para-carcinoma live tissues detected with qPCR assay; (**C**) Matrix graphs of Pearson correlations for *CCR1*, *CCR5* and *CCR7*; (**D**–**F**) ROC curves for *CCR1*, *CCR5* and *CCR7*; (**G**–**I**) survival analysis for OS in terms of *CCR1*, *CCR5* and *CCR7*; ** P<0.01; *** P<0.001.

After providing written informed consent forty-nine patients were enrolled in this research study as the validation cohort, and was named as the Guangxi cohort. The baseline information of patients in the Guangxi cohort are listed in Supplementary Table 6. The results of the IHC assay and qPCR assay both showed that the expression levels of CCR1, CCR5, and CCR7 were significantly lower in HCC tissues, compared with para-carcinoma tissues (Figure 6A, 6B). Meanwhile, it was observed that the expression levels of *CCR1*, *CCR5,* and *CCR7* were strongly correlated (Figure 6C). Additionally, *CCR1*, *CCR5,* and *CCR7* performed well for HCC diagnosis (Figure 6D–6F). In full agreement with the results in GSE14520, *CCR1* (*P*=0.02, Table 3 and Figure 6G), *CCR5* (*P*=0.017, Table 3 and Figure 6H), and *CCR7* (*P*=0.013, Table 3 and Figure 6I) were found to be significantly associated with the prognosis of HCC in the Guangxi cohort, and high levels of *CCR1*, *CCR5,* and *CCR7* expression can be used to predict a favorable prognosis. Similar results were verified in the ICGC dataset (Supplementary Figure 4), where the expression of *CCR1*, *CCR5*, and *CCR7* in tumors was higher than that in adjacent tissues, with a positive correlation. *CCR5* (*P*=0.045), and *CCR7* (*P*=0.015) were significantly associated with the prognosis of HCC in ICGC dataset.

**Table 3:** CCR1, 5, 7 were associated with OS in HCC (Cox regression).

**Gene expression**	**Patients (n=49)**	**OS**			
		**NO. of event**	**MST (months)**	**Crude HR (95% CI)**	**P**
CCR1					
Low	25	14	24	-	
High	24	15	31	0.41 (0.19-0.89)	0.024
CCR5					
Low	25	17	24	-	
High	24	12	35	0.40 (0.19-0.87)	0.017
CCR7					
Low	25	15	29	-	
High	24	14	33	0.38 (0.18-0.84)	0.013

Abbreviation: CCR, C-C chemokine receptor; OS, overall survival; NO, number; HR, hazard ratio; CI, confidence interval; MST, median survival time.

### Construction of a nomogram and prognostic signature using the Guangxi cohort

**Figure 7:**
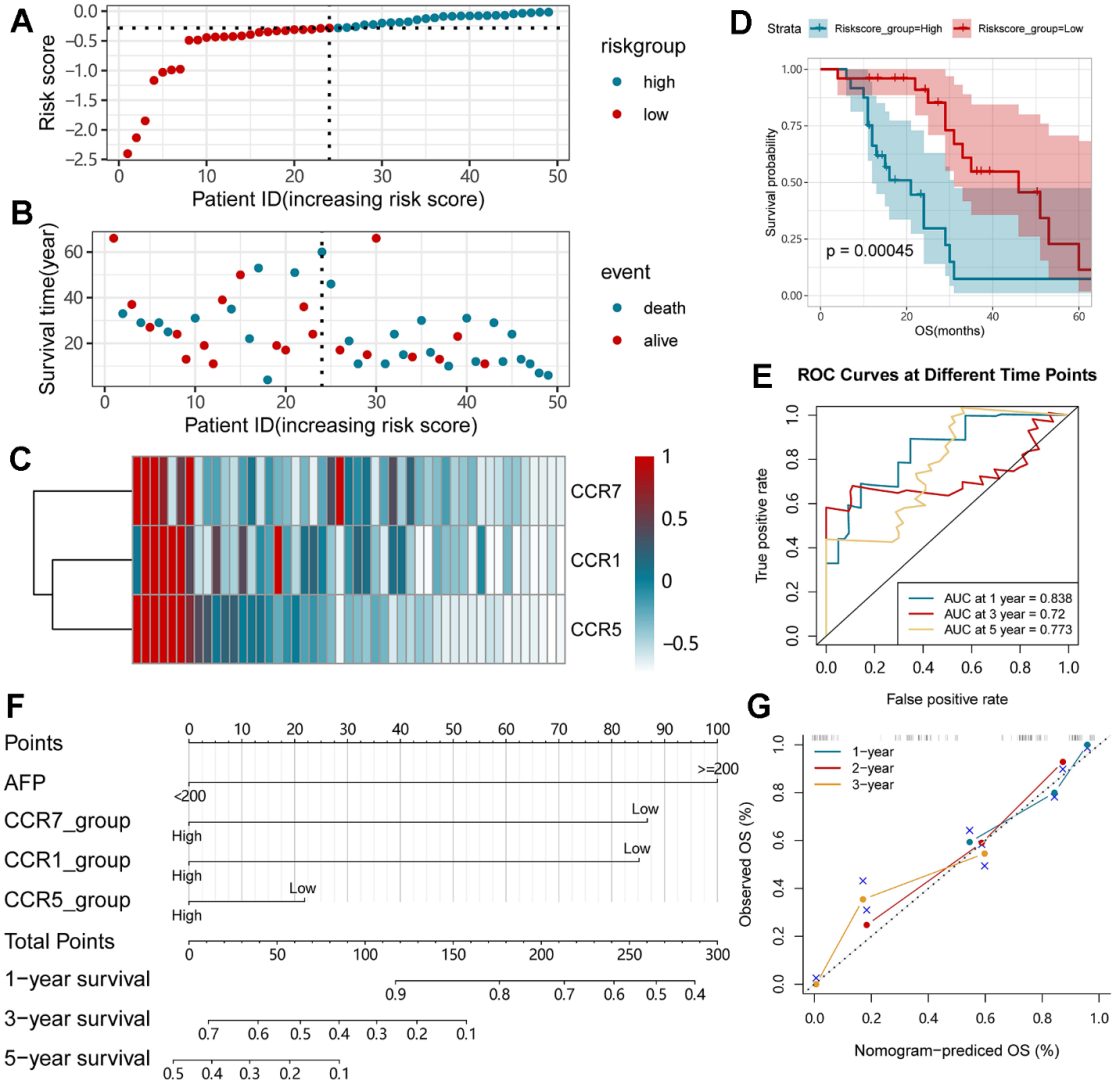
**Nomogram and the prognostic signature constructed in Guangxi cohort in terms of *CCR1*, *CCR5* and *CCR7*.** (**A**) Risk score plot; (**B**) survival status scatter plot; (**C**) heat map of the levels of expression of *CCR1*, *CCR5* and *CCR7* in low- and high-risk groups; (**D**) Kaplan-Meier curves for low- and high-risk groups; (**E**) Receiver operating characteristic curve for predicting 1-,3- or 5-year survival in HCC patients by risk score; (**F**) nomogram; (**G**) verification model for nomogram in 1-, 2- and 3-year OS respectively.

Based on the expression of *CCR1*, *CCR5* and *CCR7*, we constructed the prognostic signature and nomogram for HCC patients in the Guangxi cohort. The specific risk score formula used for the patients in the Guangxi cohort was: risk score = expression value of *CCR1* x -0.051 + expression value of *CCR5* x -0.231 + expression value of *CCR7* x -0.046. The risk score and the time of the outcome event in HCC patients of the Guangxi cohort are displayed using scatter plots (Figure 7A, 7B), and the *CCR1*, *CCR5,* and *CCR7* expression profiles of these patients are presented using a heat map (Figure 7C). We observed that patients in the high-risk group had a shorter survival compared with those in the low-risk group. The results of the survival analysis in the high and low risk groups indicated that the difference in prognosis was statistically significant (Figure 7D, *P*<0.001). The survival ROC curve indicated that the prognostic signature showed good performance in predicting 1-, 3- or 5-year OS (Figure 7E).

In the nomogram constructed using the Guangxi cohort, the parameter with the highest prognostic contribution was AFP, followed by the CCR7 (Figure 7F). The predictive power of the nomogram was assessed using the match degree between the training group and the validation group. In the nomogram of the Guangxi cohort, a high degree of superposition was observed between the self-validation cohort (color line) and training group (gray line) for the prediction of 1-, 2- or 3-year prognosis (Figure 7G).

### GSEA

**Figure 8:**
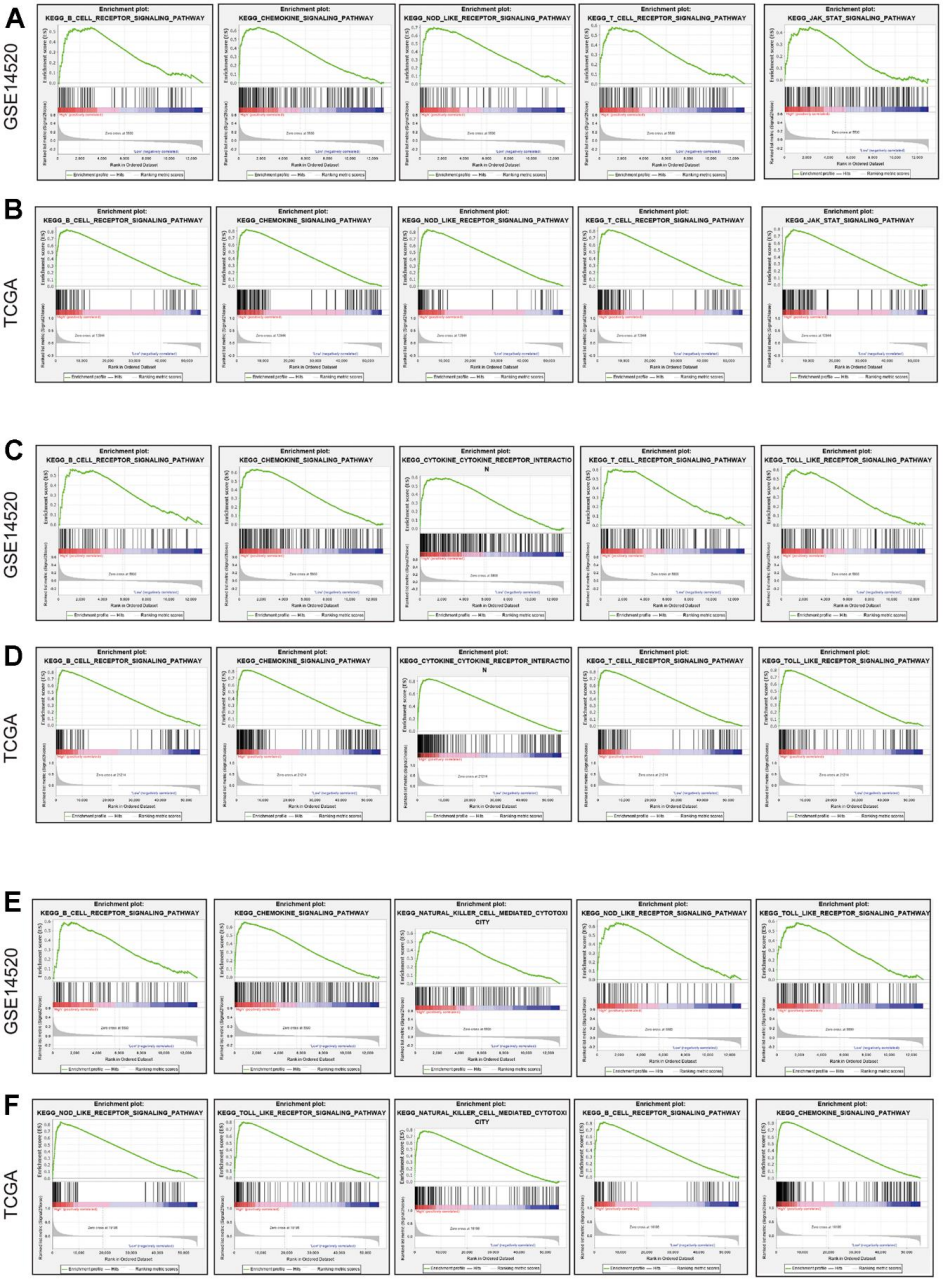
**GSEA in terms of CCR1, CCR5 and CCR7 based on C2 curated gene sets.** (**A**) Representative result of GSEA results of CCR1 in GSE14520; (**B**) representative result of GSEA results of CCR1 in TCGA; (**C**) representative result of GSEA results of CCR5 in GSE14520; (**D**) representative result of GSEA results of CCR5 in TCGA; (**E**) representative result of GSEA results of CCR7 in GSE14520; (**F**) representative result of GSEA results of CCR7 in TCGA.

After the comprehensive analysis of GSEA results in the GSE14520 dataset and the GSEA result in TCGA LIHC dataset, we observed that the enrichment results in these two datasets were very similar. Representative results are presented and reveal that *CCR1* (Figure 8A, 8B) was associated with the B cell receptor signaling pathway, chemokine signaling pathway, nod-like receptor signaling pathway, T cell receptor signaling pathway, and JAK-STAT signaling pathway. *CCR5* (Figure 8C, 8D) was associated with the B cell receptor signaling pathway, chemokine signaling pathway, cytokine-cytokine receptor signaling pathway, T cell receptor signaling pathway, and toll-like receptor signaling pathway. *CCR7* (Figure 8E, 8F) was associated with B cell receptor signaling pathway, chemokine signaling pathway, natural killer mediated cytotoxicity, nod-like receptor signaling pathway, and toll-like receptor signaling pathway. We observed that these *CCR* genes are enriched in very similar pathways in the HCC data sets, which suggests that there may be an association between them.

### Tumor-infiltrating immune cells

**Figure 9:**
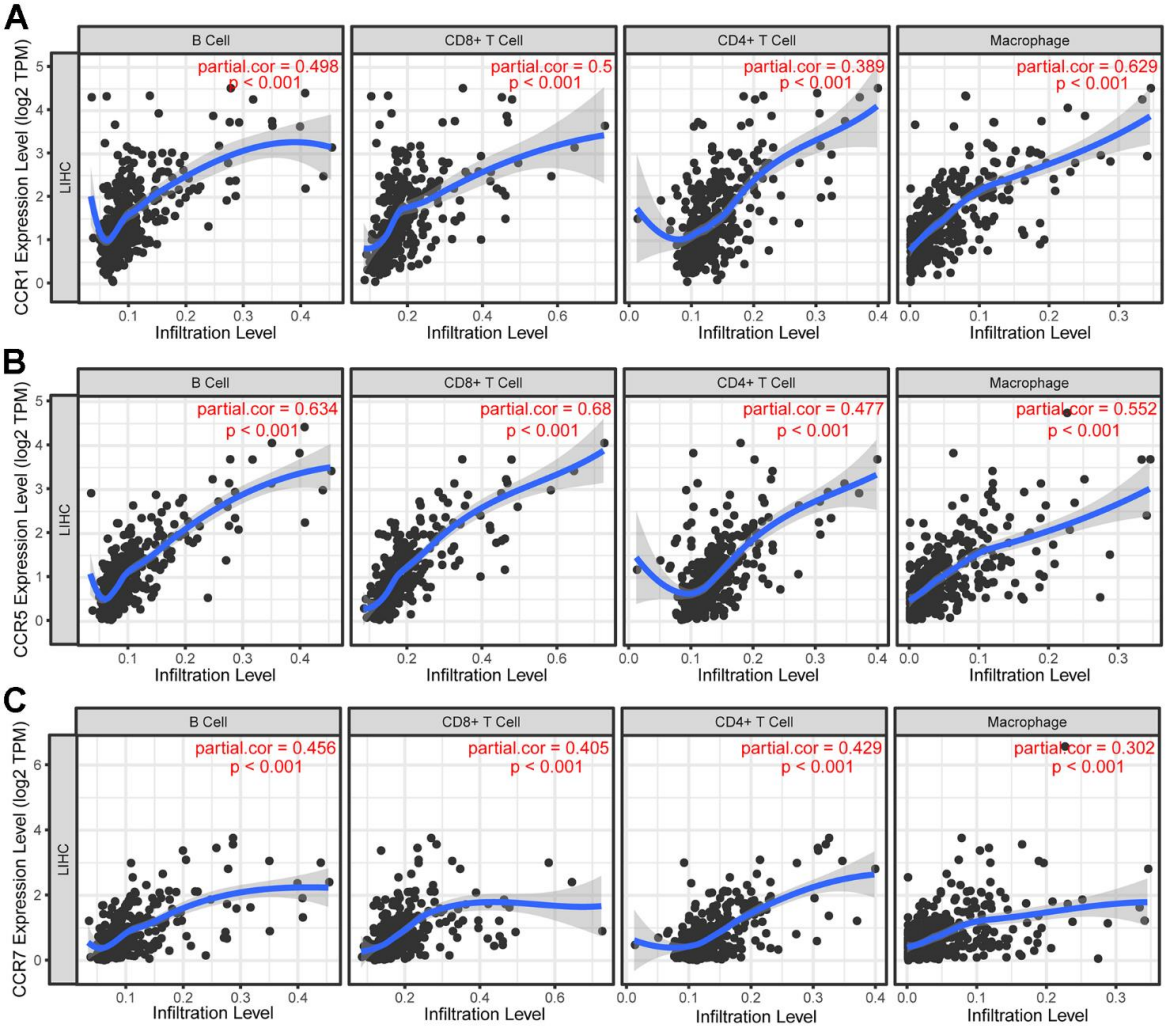
**Correlation between *CCRs* expression and tumor-infiltrating immune cells.** (**A**) Scatter plot in terms of *CCR1* expression and tumor-infiltrating immune cells; (**B**) scatter plot in terms of *CCR5* expression and tumor-infiltrating immune cells; (**C**) scatter plot in terms of *CCR7* expression and tumor-infiltrating immune cells.

TIMER is a web-based resource used to perform systematical evaluations of the clinical impact of different immune cells in diverse cancer types based on the data of TCGA database. Using TIMER, we found significant associations between CCR1, CCR5, CCR7, and immune cell infiltration in TCGA LIHC dataset. The results indicated that *CCR1* was positively correlated with the degree of B cell (Cor=0.498), CD8^+^ T cell (Cor=0.500), CD4^+^ T cell (Cor=0.389), and macrophage (Cor=0.629) infiltration in HCC tissues (Figure 9A). Additionally, we observed that *CCR5* was also positively correlated with the degree of B cell (Cor=0.634), CD8^+^ T cell (Cor=0.680), CD4^+^ T cell (Cor=0.477), and macrophage (Cor=0.552) infiltration in the HCC tissues (Figure 9B). Similarly, HCC tissues with high CCR7 expression were accompanied by a high degree of B cell (Cor=0.456), CD8^+^ T cell (Cor=0.405), CD4^+^ T cell (Cor=0.429), and macrophage (Cor=0.302) infiltration (Figure 9C).

### Biological function of CCR1, CCR5, and CCR7 in HCC

**Figure 10:**
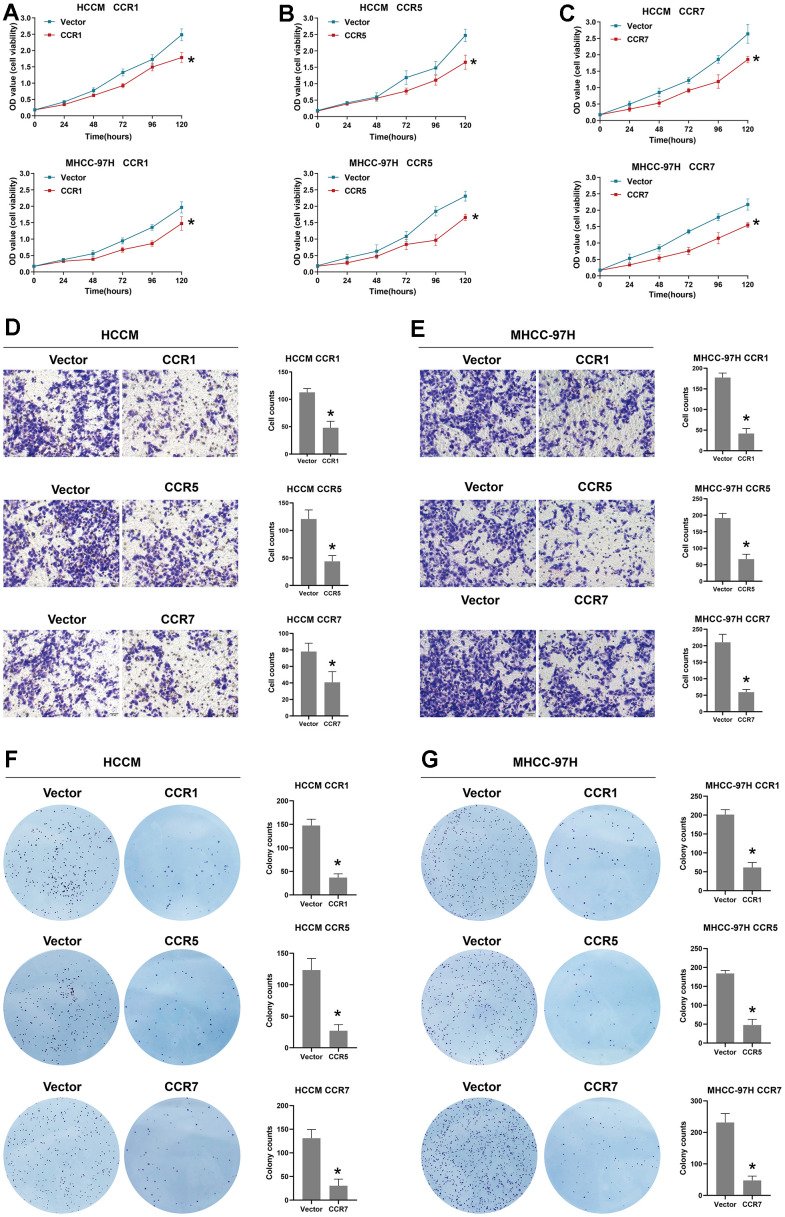
**Biological function of CCR1, CCR5 and CCR7 in HCC.** (**A**) The cell viability of between vector and CCR1 overexpression group in HCCM cells and MHCC-97 cells; (**B**) The cell viability of between vector and CCR5 overexpression group in HCCM cells and MHCC-97 cells; (**C**) The cell viability of between vector and CCR7 overexpression group in HCCM cells and MHCC-97 cells; (**D**) Representative images of Transwell assay for CCR1 overexpression, CCR5 overexpression, CCR7 overexpression and vector group in HCCM cells and corresponding histograms; (**E**) Representative images of Transwell assay for CCR1 overexpression, CCR5 overexpression, CCR7 overexpression and vector group in MHCC-97H cells and corresponding histograms. (**F**) Representative images of colony formation assay for CCR1 overexpression, CCR5 overexpression, CCR7 overexpression and vector group in HCCM cells and corresponding histograms; (**G**) Representative images of colony formation assay for CCR1 overexpression, CCR5 overexpression, CCR7 overexpression and vector group in MHCC-97H cells and corresponding histograms.

The above findings indicate that CCR1, CCR5, and CCR7 play important roles in the development of HCC based on their close association with prognosis. Therefore, we explored the biological functions of CCR1, CCR5, and CCR7 inducing the upregulation of these genes in HCC cells. All functional assays were performed on two HCC cell lines, MHCC-97 and HCCM. The CCK-8 assay results indicated that CCR1, CCR5, and CCR7 upregulation inhibited the growth viability of the HCC cells (Figure 10A–10C). The results of the Transwell assay indicated that CCR1, CCR5, and CCR7 upregulation limited the migration ability of HCC cells, which also suggests that CCR1, CCR5, and CCR7 play a role in HCC metastasis (Figure 10D, 10E). The results of the colony formation assays showed that the overexpression of CCR1, CCR5, and CCR7 inhibited the ability of cells to be cloned into spheres, indicating that CCR1, CCR5, and CCR7 affect the stemness of HCC (Figure 10F, 10G).

## DISCUSSION

Due to the high incidence and fatality rate of HCC, the disease has brought great suffering to patients. Early diagnostic biomarkers and prognostic biomarkers of HCC are urgently need to be identified for the prevention and treatment of HCC. During recent years, achievements in immune research have made great breakthroughs in HCC treatment. It has been demonstrated that *CCRs,* which are chemokine receptors, play crucial roles in immunity and inflammation, but only a few reports have been published on *CCRs* in HCC. In this investigation, we inspected the clinical significance of members of the CCR gene family using TCGA LIHC dataset and the GSE14520 dataset to explore the potential mechanisms of CCR genes in HCC using bioinformatics tools.

First, we screened for genes that were differentially expressed between HCC and para-carcinoma tissue. The differentially expressed genes in TCGA LIHC dataset and GSE14520 dataset did not completely overlap, possibly due to ethnic inconsistencies of patients included in the two datasets. Hepatocellular carcinoma patients in the GSE14520 dataset were all Chinese, while HCC patients in the TCGA data set were mainly Caucasian. However, we found common results between the two datasets. We observed that the expression levels of *CCR1*, *CCR2*, *CCR5,* and *CCR7* were significantly lower in the HCC tissues in TCGA LIHC dataset and GSE14520 dataset, compared with para-carcinoma tissues.

Furthermore, survival analysis of TCGA and GSE14520 datasets showed that *CCR1*, *CCR5*, and *CCR7* were all significantly associated with the OS of HCC patients. Integral analysis, nomogram, and the prognostic model created based on *CCR1*, *CCR5*, and *CCR7* all showed good performance for the prognostic evaluation of HCC. It must be noted that high CCR1 expression in the GSE14520 dataset was associated with a positive outcome, whereas high CCR1 expression in TCGA was associated with a poor prognosis. We further examined the prognostic significance of *CCR1*, *CCR5,* and *CCR7* in patients with HCC in the Guangxi and ICGC cohort. A similar trend was observed with the GSE14520 dataset. Hepatitis B virus exposure is the main cause of HCC in China [38], while NAFLD is the main cause of HCC in the United States of America [39]. We hypothesized that *CCR1* may play distinct roles in HCC based on pathogenesis.

We reviewed reports on *CCR1* in multiple cancers, which showed that the higher expression of CCR1 was correlated with a better prognosis of head and neck cancer, ovarian cancer and melanoma [40]. Whereas certain other reports showed that higher CCR1 expression was accompanied by a worse outcome of glioma, lung cancer, renal cancer, and testicular cancer [41]. Zhu et al. found that CCL14 could induce the apoptosis of hepatocellular carcinoma cells by activating CCR1 [42], and supports the conclusion we obtained using the GSE14520 dataset and Guangxi cohorts. It was also found that CCL15 induces HCC cell migration and invasion through the activation of CCR1, leading to a worse prognosis [43]. CCR1 has many ligands, which include CCL2, CCL3, CCL4, CCL5, CCL7, CCL8, CCL14, CCL15, CCL16, and CCL23. There are differences in the chemokine levels of individuals of different backgrounds, and may lead to radically different outcomes following CCR1 activation. Some studies have claimed that CCR1 promotes NK cell infiltration, while it has also been reported that CCR1 activation reduces immune infiltration and halts the progression of pancreatic intraepithelial neoplasia [44]. Additionally, elevated CCL16 (ligand of CCR1) expression exerted anticancer effects in mice with breast [42, 45], colon [42], and prostate cancers [46]. The researchers found that this anti-cancer effect was due to an increase in CD4+ T cell, CD8+ T cell, and DC cell infiltration into tumors [42, 46].

CCR5 is usually considered as the HIV specific binding site on the surface of T cells. CCR5 expression has been reported to be associated with the growth of multiple cancers, including breast cancer, ovarian cancer, cervical cancer, prostate cancer, colon cancer, melanoma, Hodgkin’s lymphoma, and multiple myeloma [47]. Wang et al. found that the activation of the CCL4/CCR5 axis significantly induced γδ T-cell infiltration in HCC, thereby improving the prognosis of HCC patients. Leronlimab (PRO140) is a humanized IgG4 monoclonal antibody that targets chemokine receptor 5 (CCR5). It has been demonstrated to block tumor metastasis in invasive breast and prostate cancers in both cell and animal models [48]. It has also been shown that CCR5 can activate CD1d+ NKT cells, while also being able to promote altered NK cell infiltration, indicating that chemokines not only affect the attractiveness but also the function of immune cells [49]. Moreover, the CCL5/CCR5 axis can also induce the accumulation of anti-cancer tumor-infiltrating lymphocytes (TIL) in tumors, which increases their cytotoxicity [50–54]. The CCL5/CCR5 axis is also responsible for recruiting NK cells and T helper cells type 1 (Th1) to infiltrate tumors [51–54]. Although the function of CCR5 in HCC remains to be elucidated, it has been found to be associated with chronic liver inflammation caused by a variety of pathogens and may be involved in the occurrence and development of HCC [55, 56]. The Human Protein Atlas (https://www.proteinatlas.org/) demonstrated that patients with CCR5 upregulation have shown a better outcome in various cancers, including thyroid, lung, colorectal, head and neck, stomach, liver, prostate, breast, and cervical cancers. However, CCR5 was found to be associated with a poor prognosis in several other cancers, such as glioma, kidney cancer, and testicular cancer [40, 41]. Therefore, the function of CCR5 may validate the prognostic value of CCR5 in multiple cancers.

Hypoxia and prostaglandin E2 increase the expression of CCR7 in cancer cells, thereby affecting cell stemness and proliferation potential [15, 57–61]. In colorectal cancer cells, CCL19 activates CCR7, thereby inducing miR-206 upregulation, which suppresses angiogenesis to inhibit the ERK/MAPK-HIF-1-VEGF pathway [62]. To our knowledge, this is the first study to inspect the prognostic significance of CCR7 in HCC using multiple datasets. CCR7 was found to be strongly associated with a better outcome in hepatocellular carcinoma patients.

GSEA results of *CCR1*, *CCR5*, and *CCR7* were very similar to each other, and all three were found to be associate with the chemotactic function of B cells and T cells. Subsequently, we investigated the correlation between *CCR1*, *CCR5*, and *CCR7* and the degree of immune cell infiltration in the tumor microenvironment. The results are consistent with the results obtained using GSEA. We observed that *CCR1*, *CCR5*, and *CCR7* were positively correlated with the degree of B cell, CD8+ T cell, CD4+ T cell, and macrophage infiltration in HCC tissues.

There are several limitations in this investigation. The sample size of the Guangxi cohort included in this investigation was small, and a larger sample size will lead to more reliable results. This study primarily discussed the diagnostic and prognostic value of *CCR* genes in HCC. However, the function of diagnostic and prognostic biomarkers in HCC needs to be verified further. We found that *CCR1*, *CCR5, CCR7* were associated with B cell, CD8+ T cell, CD4+ T cell, and macrophage infiltration in HCC tissues. However, the mechanism by which they cause leukocyte enrichment is still unclear, and animal experiments may need to be conducted.

## CONCLUSIONS

It was found that the expressions of *CCR1*, *CCR5,* and *CCR7* are associated with the OS of HCC patients. *CCRs* were found to be closely associated with several signaling pathways, such as the B cell receptor signaling pathway, chemokine signaling pathway, and T cell receptor signaling pathway. Additionally, we found that *CCR1*, *CCR5,* and *CCR7* expression levels were significantly positively correlated with the degree of immune infiltration of B cells, CD8^+^ T cells, CD4^+^ T cells, and macrophages. Therefore, our results suggest that *CCR1*, *CCR5,* and *CCR7* are crucial prognostic biomarkers of HCC, which may be involved in HCC by inducing immune cell infiltration.

## Supplementary Material

Supplementary Figures

Supplementary Tables
