# Supplementary material for: Role of CCR1/5/7 in hepatocellular carcinoma: a study on prognostic evaluation, molecular subtyping, and association with immune infiltration
Source: Aging (Albany NY). 2024 Mar 28;16(7):6229–61. doi: 10.18632/aging.205698 (PMC11042939; doi:10.18632/aging.205698)
Supplement: Supplementary Figures [file aging-16-205698-s001.pdf]

SUPPLEMENTARY FIGURES

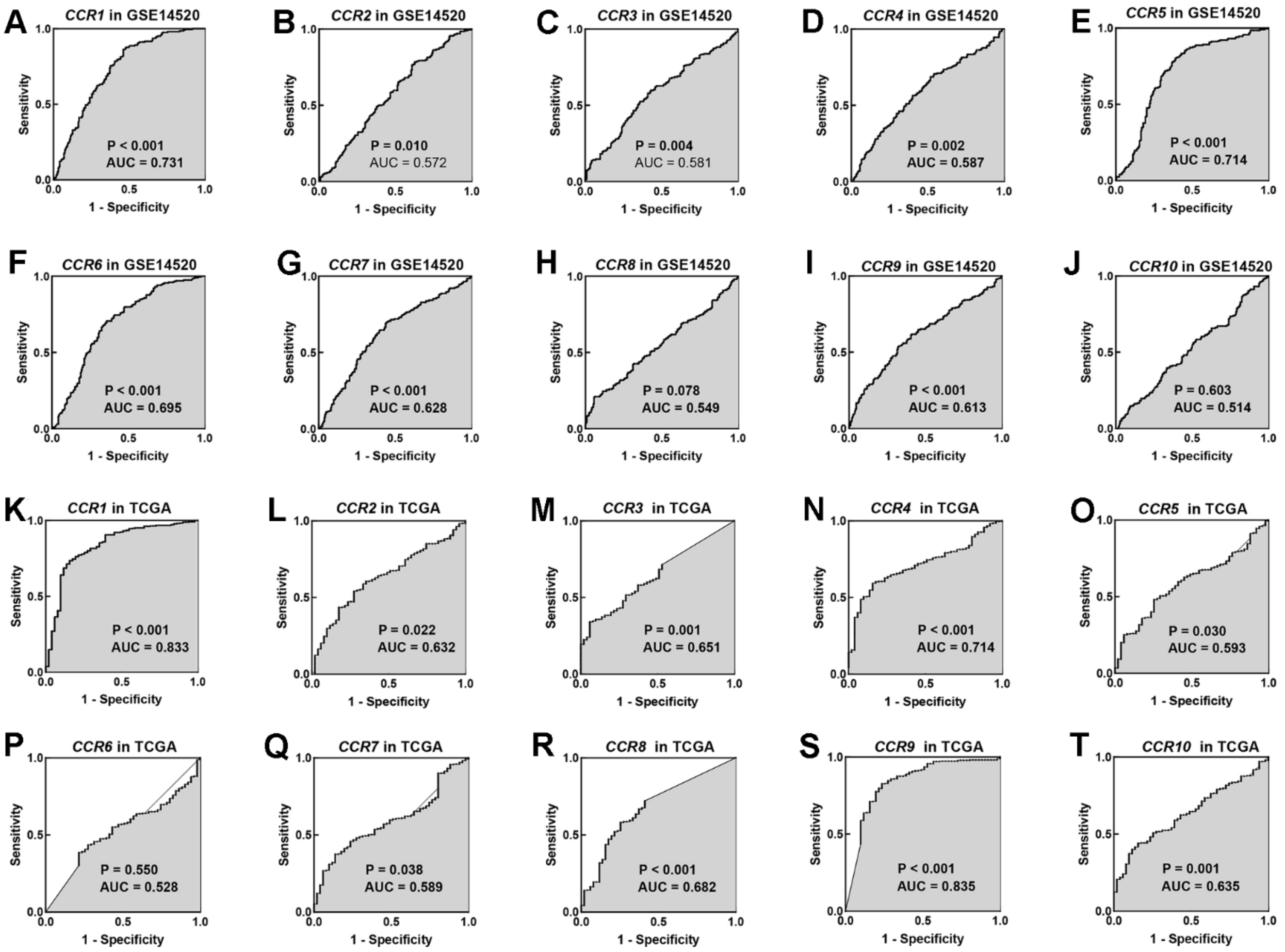

Supplementary Figure 1. ROC curves of CCRs in GSE14520 dataset and TCGA database. (**A**–**J**) *CCR1-CCR10* in GSE14520; (**K**–**T**) *CCR1-CCR10* in TCGA.

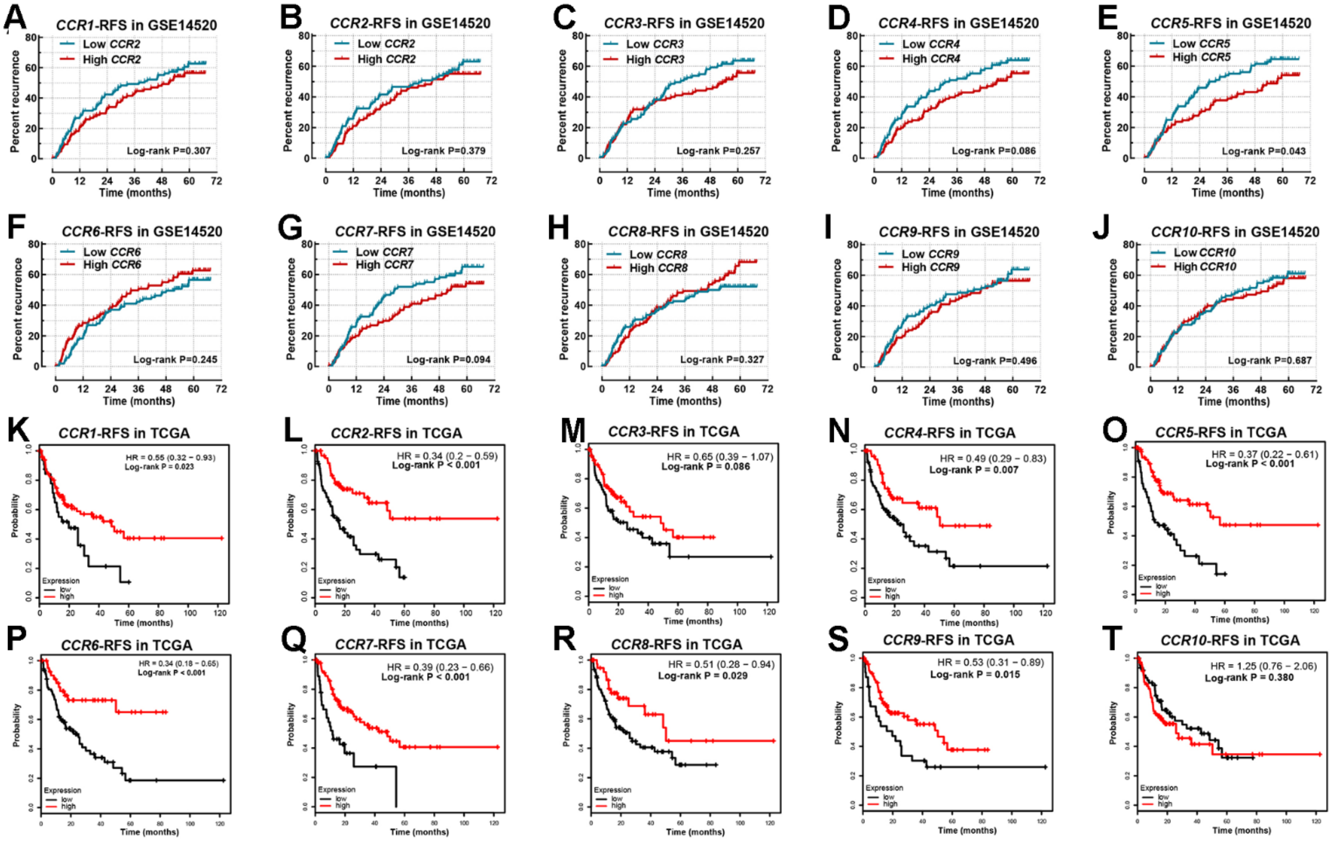

Supplementary Figure 2. Survival analysis for RFS in GSE14520 dataset and TCGA database. (**A**–**J**) *CCR1-CCR10* in GSE14520; (**K**–**T**) *CCR1-CCR10* in TCGA.

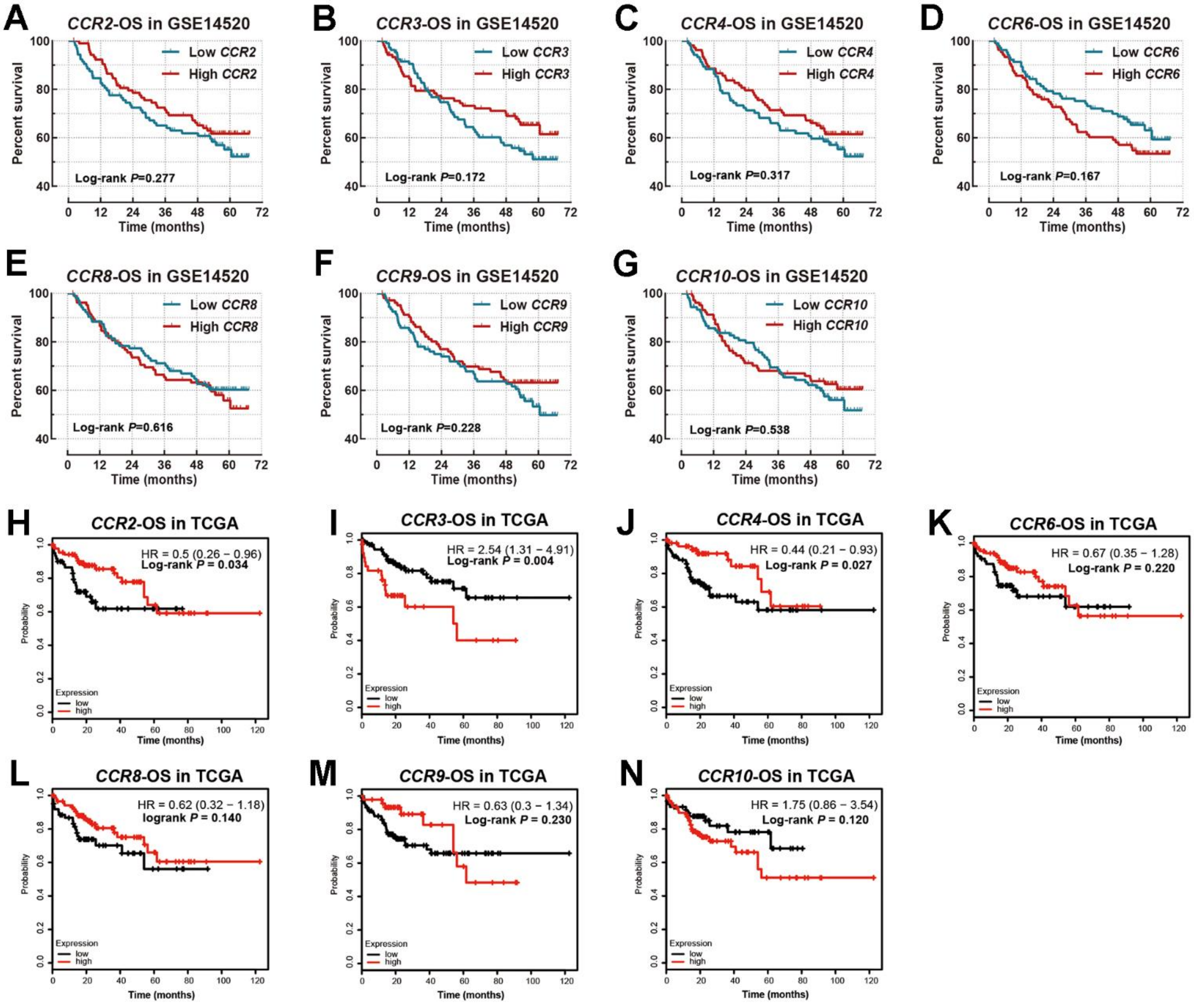

Supplementary Figure 3. Survival curves for OS in GSE14520 dataset and TCGA database. (**A**–**G**) CCR2, CCR3, CCR4, CCR6, CCR8, CCR9 and CCR10 in GSE14520; (**H**–**N**) CCR2, CCR3, CCR4, CCR6, CCR8, CCR9 and CCR10 in TCGA database.

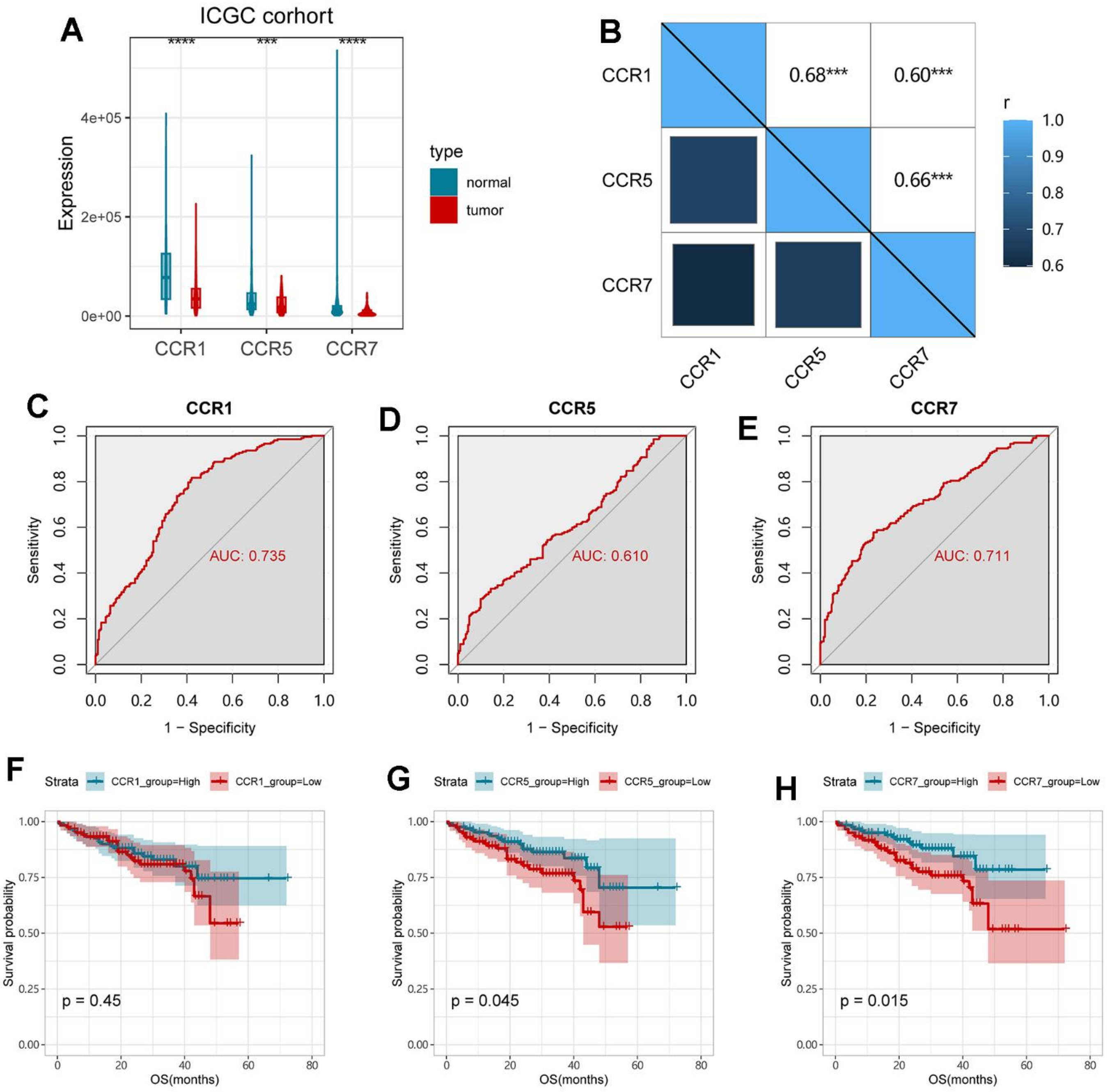

Supplementary Figure 4. Validation of the clinical significance of CCR1, CCR5, and CCR7 in the ICGC cohort. (**A**) Expression level of *CCRs* between HCC and para-carcinoma tissues in ICGC; (**B**) Matrix graphs of Pearson correlations for *CCR1*, *CCR5* and *CCR7*; (**C**–**E**) ROC curves for *CCR1*, *CCR5* and *CCR7*; (**F**–**H**) survival analysis for OS in terms of *CCR1*, *CCR5* and *CCR7*; ** P<0.01; *** P<0.001.
