# Supplementary material for: Role of CCR1/5/7 in hepatocellular carcinoma: a study on prognostic evaluation, molecular subtyping, and association with immune infiltration
Source: Aging (Albany NY). 2024 Mar 28;16(7):6229–61. doi: 10.18632/aging.205698 (PMC11042939; doi:10.18632/aging.205698)
Supplement: Supplementary Tables [file aging-16-205698-s002.pdf]

SUPPLEMENTARY TABLES

Supplementary Table 1. Function annotation and pathway enrichment result of CCR genes in DAVID.

Category	Term	P-Value	Genes
GOTERM_BP_DIRECT	GO:0006935~chemotaxis	0.000000000000000000041784129592	CCR9, CCR8, CCR7, CCR6, CCR5, CCR4, CCR1, CCR3, CCR2, CCR10
GOTERM_BP_DIRECT	GO:0007204~positive regulation of cytosolic calcium ion concentration	0.000000000000000000099937473494	CCR9, CCR8, CCR7, CCR6, CCR5, CCR4, CCR1, CCR3, CCR2, CCR10
GOTERM_BP_DIRECT	GO:0070098~chemokine-mediated signaling pathway	0.000000000000000000609453560723	CCR9, CCR8, CCR6, CCR5, CCR4, CCR1, CCR3, CCR2, CCR10
GOTERM_BP_DIRECT	GO:0006955~immune response	0.000000000000128743921 7957590000	CCR9, CCR8, CCR7, CCR6, CCR5, CCR4, CCR1, CCR2, CCR10
GOTERM_BP_DIRECT	GO:0002407~dendritic cell chemotaxis	0.000000000009027312906 1108200000	CCR7, CCR6, CCR5, CCR1, CCR2
GOTERM_BP_DIRECT	GO:0006968~cellular defense response	0.000000002093300687356 2200000000	CCR9, CCR6, CCR5, CCR3, CCR2
GOTERM_BP_DIRECT	GO:0007186~G-protein coupled receptor signaling pathway	0.000000004035840771988 6700000000	CCR9, CCR8, CCR7, CCR6, CCR5, CCR4, CCR2, CCR10
GOTERM_BP_DIRECT	GO:0006954~inflammatory response	0.000000667232558404180000000000	CCR7, CCR5, CCR4, CCR1, CCR3, CCR2
GOTERM_BP_DIRECT	GO:2000510~positive regulation of dendritic cell chemotaxis	0.003746427721986910000000000000	CCR7, CCR6
GOTERM_BP_DIRECT	GO:0048872~homeostasis of number of cells	0.006947715932952390000000000000	CCR7, CCR4
GOTERM_BP_DIRECT	GO:0090026~positive regulation of monocyte chemotaxis	0.008544928453991170000000000000	CCR1, CCR2
GOTERM_BP_DIRECT	GO:0007155~cell adhesion	0.023632805453578200000000000000	CCR8, CCR1, CCR3
GOTERM_BP_DIRECT	GO:0019722~calcium-mediated signaling	0.027011068117087800000000000000	CCR6, CCR5
GOTERM_BP_DIRECT	GO:0009611~response to wounding	0.033271565460816100000000000000	CCR1, CCR2
GOTERM_BP_DIRECT	GO:0006816~calcium ion transport	0.040013341414467300000000000000	CCR5, CCR1
GOTERM_BP_DIRECT	GO:0006874~cellular calcium ion homeostasis	0.048766437521694700000000000000	CCR1, CCR2
GOTERM_CC_DIRECT	GO:0005887~integral component of plasma membrane	0.000000001087206582097 7600000000	CCR9, CCR8, CCR6, CCR5, CCR4, CCR1, CCR3, CCR2, CCR10
GOTERM_CC_DIRECT	GO:0005886~plasma membrane	0.000001535648146166550000000000	CCR9, CCR8, CCR7, CCR6, CCR5, CCR4, CCR1, CCR3, CCR2, CCR10
GOTERM_CC_DIRECT	GO:0009986~cell surface	0.000086558266950133400000000000	CCR9, CCR7, CCR6, CCR5, CCR10
GOTERM_CC_DIRECT	GO:0009897~external side of plasma membrane	0.000125552792876137000000000000	CCR7, CCR5, CCR4, CCR1
GOTERM_CC_DIRECT	GO:0016021~integral component of membrane	0.002981401570357240000000000000	CCR9, CCR7, CCR5, CCR4, CCR1, CCR3, CCR2, CCR10
GOTERM_MF_DIRECT	GO:0016493~C-C chemokine receptor activity	0.000000000000000000000000000001	CCR9, CCR8, CCR7, CCR6, CCR5, CCR4, CCR1, CCR3, CCR2, CCR10
GOTERM_MF_DIRECT	GO:0004950~chemokine receptor activity	0.000000000000000000009033295325	CCR9, CCR8, CCR6, CCR5, CCR4, CCR1, CCR3, CCR2
GOTERM_MF_DIRECT	GO:0019957~C-C chemokine binding	0.000003785931735710800000000000	CCR6, CCR5, CCR1
GOTERM_MF_DIRECT	GO:0071791~chemokine (C-C motif) ligand 5 binding	0.001066034867444990000000000000	CCR5, CCR1
GOTERM_MF_DIRECT	GO:0004435~phosphatidylinositol phospholipase C activity	0.014306498827950600000000000000	CCR5, CCR1
GOTERM_MF_DIRECT	GO:0015026~coreceptor activity	0.015884824028914600000000000000	CCR8, CCR5
KEGG_	hsa04062:Chemokine signaling pathway	0.0000000000000000637962	CCR9, CCR8, CCR7, CCR6, CCR5, CCR4,

PATHWAY		3361818630	CCR1, CCR3, CCR2, CCR10
KEGG_ PATHWAY	hsa04060:Cytokine-cytokine receptor interaction	0.00000000000007411377 6970328900	CCR9, CCR8, CCR7, CCR6, CCR5, CCR4, CCR1, CCR3, CCR2, CCR10
KEGG_ PATHWAY	hsa05203:Viral carcinogenesis	0.00191832737624775000 0000000000	CCR8, CCR5, CCR4, CCR3

Abbreviation: CCR, C-C motif chemokine receptor; DAVID, Database for Annotation, Visualization and Integrated Discovery; GO, Gene Ontology; BP, biological process; CC, cellular component; MF, molecular function.

Supplementary Table 2. Basic characteristics of 212 HCC patients.

Variables	Patients (n=212)	RFS				OS			
		No. of events	MST (months)	HR (95% CI)	*P*	No. of events	MST (months)	HR (95% CI)	*P*
Age(years)									
≤60	175	96	45.9	1		69	NA	1	
>60	37	20	48	0.974(0.602-1.578)	0.916	13	NA	0.8643(0.478-1.564)	0.629
Gender									
Female	29	10	NA	1		8	NA	1	
Male	183	106	40.1	2.143(1.120-4.100)	0.018	74	NA	1.704(0.821-3.534)	0.148
Multinodular									
Single	167	90	49.1	1		59	NA	1	
Multiple	45	26	28.7	1.216(0.785-1.883)	0.381	23	47.9	1.607(0.992-2.604)	0.052
Tumor size									
≤5 cm	137	73	51.1	1		46	NA	1	
>5 cm	74	43	28.4	1.409(0.966-2.056)	0.073	36	53.3	1.975(1.274-3.060)	0.002
Cirrhosis									
NO	17	5	NA	1		2	NA	1	
YES	195	111	37.9	2.612(1.066-6.402)	0.029	80	NA	4.335(1.065-17.638)	0.025
BCLC									
0	20	6	NA	1		2	NA	1	
A	143	74	51.6	2.050(2.892-4.711)	0.091	48	NA	4.119(1.001-16.951)	0.05
B	22	15	26.9	4.019(1.550-10.421)	0.004	12	46.1	8.992(2.005-40.320)	0.004
C	27	21	8.9	6.163(2.477-15.333)	<0.001	20	13.6	18.993(4.419-81.632	<0.001
Serum AFP									
≤300 ng/ml	115	62	48	1		39	NA	1	
>300 ng/ml	94	54	35.2	1.200(0.833-1.728)	0.327	43	NA	1.546(1.002-2.385)	0.049
TNM stage									
I+II	165	83	53	1		52	NA	1	
III	47	33	18	2.279(1.517-3.423)	<0.001	30	18	3.425(2.171-5.405)	<0.001

Abbreviation: HCC, hepatocellular carcinoma; No., number; MST, middle survival time; HR, hazard ratio.

Supplementary Table 3. Correlation between CCR1 expression and clinicopathologic.

Variables	Patients (n=212)	CCR1 expression		X^2	P-value
		Low	High		
Age(years)					
≤60	175	88	87		
>60	37	18	19	0.033	0.856
Gender					
Female	29	13	16		
Male	183	93	90	0.36	0.549
Multinodular					
Single	167	83	84		
Multiple	45	23	22	0.028	0.867
Serum AFP					
>300ng/ml	94	44	50		
≤300ng/ml	115	61	54	1.142	0.565
Cirrhosis					
No	17	10	7		
Yes	195	96	99	0.576	0.448
BCLC					
0	20	10	10		
A	143	68	75		
B	22	12	10		
C	27	16	11	1.45	0.694
Tumor size					
≤5cm	137	72	65		
>5cm	74	34	40	1.844	0.398
TNM stage					
I+II	165	80	85		
III	47	26	21	0.683	0.408

Supplementary Table 4. Correlation between CCR5 expression and clinicopathologic.

Variables	Patients (n=212)	CCR5		X^2	P-value
		Low	High		
Age(years)					
≤60	175	88	87		
>60	37	18	19	0.033	0.856
Gender					
Female	29	13	16		
Male	183	93	90	0.36	0.549
Multinodular					
Single	167	79	88		
Multiple	45	27	18	2.285	0.131
Serum AFP					
>300ng/ml	94	50	44		
≤300ng/ml	115	55	60	0.934	0.627
Cirrhosis					
No	17	8	9		
Yes	195	98	97	0.064	0.800
BCLC					
0	20	7	13		
A	143	69	74		
B	22	15	7		
C	27	15	12	5.217	0.157
Tumor size					
≤5cm	137	59	78		
>5cm	74	46	28	8.013	0.018
TNM stage					
I+II	165	79	86		
III	47	27	20	1.34	0.247

Supplementary Table 5. Correlation between CCR7 expression and clinicopathologic.

Variables	Patients (n=212)	CCR7		X^2	P-value
		Low	High		
Age(years)					
≤60	175	87	88		
>60	37	19	18	0.033	0.856
Gender					
Female	29	12	17		
Male	183	94	89	0.999	0.318
Multinodular					
Single	167	84	83		
Multiple	45	22	23	0.028	0.867
Serum AFP					
>300ng/ml	94	42	52		
≤300ng/ml	115	62	54	4.49	0.106
Cirrhosis					
No	17	8	9		
Yes	195	98	97	0.064	0.800
BCLC					
0	20	6	14		
A	143	72	71		
B	22	11	11		
C	27	17	10	5.022	0.170
Tumor size					
≤5cm	137	60	77		
>5cm	74	45	29	1.844	0.398
TNM stage					
I+II	165	78	87		
III	47	28	19	2.214	0.137

Supplementary Table 6. Basic characteristics of 49 HCC patients in Guangxi cohort (Cox regression).

Variables	Patients No. (%)	Overall survival	
		Crude HR (95% CI)	P
Age(years)			
<60	41 (83.7)	-	
>=60	8 (16.3)	0.95 (0.33-2.78)	0.928
Gender			
Male	28 (57.1)	-	
Female	21 (42.9)	1.52 (0.69-3.31)	0.296
BMI			
<24	32 (65.3)	-	
>=24	17 (34.7)	0.63 (0.28-1.42)	0.263
Smoking			
Yes	13 (26.5)	-	
No	36 (73.5)	1.17 (0.47-2.88)	0.733
Drinking			
Yes	11 (22.4)	-	
No	38 (77.6)	0.92 (0.35-2.46)	0.871
Cirrhosis			
Yes	37 (75.5)	-	
No	12 (24.5)	1.77 (0.78-4.03)	0.172
BCLC stage			
A	36 (73.5)	-	
B+C	13 (26.5)	0.75 (0.32-1.78)	0.518
AFP			
<200	30 (61.2)	-	
>=200	19 (38.8)	2.97 (1.34-6.58)	0.007
Tumor size			
<5	32 (65.3)	-	
>=5	17 (34.7)	1.47 (0.69-3.12)	0.321
Histological grade			
G1	4 (8.2)	-	
G2	29 (59.2)	0.75 (0.25-2.27)	0.608
G3	16 (32.7)	1.20 (0.35-4.11)	0.774
MVI			
M0	34 (69.4)	-	
M1+M2	15 (30.6)	0.41 (0.16-1.05)	0.062

Abbreviation: HCC, hepatocellular carcinoma; No., number; MVI, microvascular invasion; HR, hazard ratio.
